# Mucociliary Clearance Inspired Nanozyme‐centric Hydrogel Composites for Integrated Bacterial Detection

**DOI:** 10.1002/advs.202503809

**Published:** 2025-07-21

**Authors:** Zhaoling Tan, Xuejiao Wang, Zhaoyue Wang, Jieke Jiang, Xi Yao

**Affiliations:** ^1^ Department of Biomedical Sciences City University of Hong Kong Kowloon Hong Kong SAR 999077 P. R. China; ^2^ Shenzhen Research Institute of City University of Hong Kong Shenzhen 518057 P. R. China; ^3^ Medicine center for PanorOmic Sciences University of Hong Kong Pokfulam Hong Kong SAR P. R. China; ^4^ Department of Thermal and Fluid Engineering University of Twente Enschede 7500AE The Netherlands

**Keywords:** bacteria biosensing, hydrogel, nanowire, nanozyme, point‐of‐care detection

## Abstract

The rapid movement of populations has led to the transmission and infection of pathogens, imposing a significant burden on global healthcare systems and economies. Despite considerable progress, current strategies heavily rely on specialized instruments or complex processing of bacterial samples. Inspired by pulmonary cilia and the mucus on it, a nanozyme‐centric hydrogel network, integrated into nanowires for comprehensive bacterial lysis and detection. Nanozymes serve as multifunctional initiators and crosslinkers for the hydrogel network, enabling a one‐step gelation process that addresses the aggregation issues of nanozyme particles, and improving the sensitivity of colorimetric detection. By integrating the nanozyme‐centric hydrogel with the rapid piercing capabilities of the nanowire arrays, an efficient platform is developed for the disinfection, lysis, and detection of various bacterial strains. Moreover, this detection platform is adaptable to flexible substrates and can be integrated with machine learning algorithms, promoting versatile home‐based pathogen testing in resource‐limited settings.

## Graphical abstract

Inspired by human pulmonary cilia and their mucus defend against bacteria, nanozyme‐centric hydrogel networks have been assembled into nanowires, combining the rapid penetration capability of the nanowire arrays with the adhesion and detection functions of the nanozyme‐centric hydrogel networks. This creates an enhanced method for disinfection, lysis, and detection of various bacterial strains.

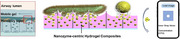

## 1 Introduction

Population movements exacerbate pathogen infections, while diagnostic delays in developing regions impose a significant strain on healthcare systems.^[^ ^1^ ^]^ Patient self‐testing is widely recognized as an ideal method, necessitating stringent standards for both safety and user‐friendliness. Point‐of‐care testing provides a straightforward method^[^ ^2^ ^]^ but mainly targets live pathogens^[^ ^3^ ^]^ due to challenges (lysis buffers^[^ ^4^ ^]^ or high fluid pressure^[^ ^5^ ^]^) of integrating bacterial lysis techniques. To facilitate in‐home monitoring, there is a pressing need for more easily integrated lysis methods. Sharp nanostructures can effectively penetrate bacterial cells through mechanical forces^[^ ^6^ ^]^ and are easy to fabricate or pattern on various substrates,^[^ ^7^ ^]^ facilitating their seamless integration into miniaturized devices for biosensing applications.^[^ ^8^ ^]^ On this basis, to facilitate patient self‐testing, a highly efficient and portable component of signal transduction must be integrated into the system after lysis to obtain sensing signals.

Biological enzymes, such as horseradish peroxidase (HRP), have been widely employed in various sensing methods for signal transduction amplification, effectively converting chemical signals into recognizable outputs.^[^ ^9^ ^]^ The high cost and inherent fragility of biological enzymes^[^ ^10^ ^]^ have promoted the development of artificial nanozymes^[^ ^11^ ^]^ that possess similar catalytic activity and enhanced stability.^[^ ^12^ ^]^ Among these, Cu₂O nanoparticles are known for their simple synthesis, low cost, good biocompatibility, and antibacterial properties, as well as their excellent HRP‐mimetic activity,^[^ ^13^ ^]^ generating diverse outputs in sensing applications,^[^ ^14^ ^]^ including colorimetric,^[^ ^15^ ^]^ fluorescent,^[^ ^16^ ^]^ and electrochemical signals.^[^ ^17^ ^]^ Nonetheless, nanoparticle aggregation^[^ ^18^ ^]^ is a common issue in these applications that can adversely impact detection sensitivity, highlighting the need for effective encapsulation strategies to enhance catalytic efficiency and portable detection methods. Hydrogels are frequently chosen as encapsulation materials because of their transparency and biocompatibility,^[^ ^19^ ^]^ but the weak interactions between hydrogel and nanomaterials can lead to leakage during repeated sample loading and washing. Furthermore, the use of additional initiators or strong oxidants for polymerization of the hydrogel may interfere with the chemical structure and enzymatic properties of nanomaterials. Therefore, robust and straightforward encapsulation methods are urgently needed that can effectively prevent leakage while maintaining enzyme activity.

**Figure 1:**
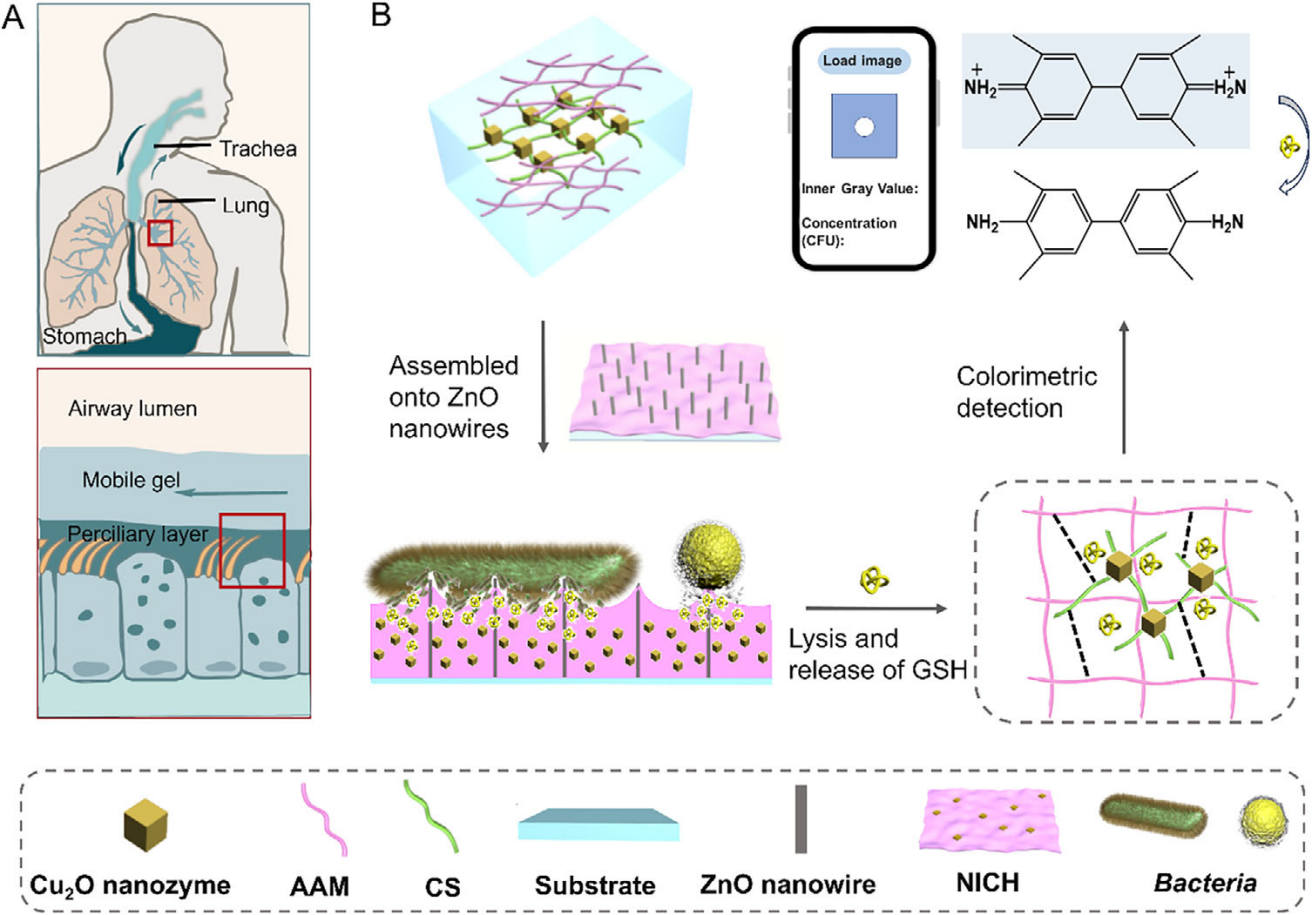
Schematic overview of nanozymes (Cu₂O) – initiated and crosslinked hydrogel (NICH) and its assembly onto nanowires with bioinspired lung cilia and mucus A) characteristics for bacterial cleavage and detection B). First, Cu₂O nanoparticles initiate the polymerization of acrylamide (AAM) monomers through free radical generation, using N, N'‐Methylenebisacrylamide (MBAA) as a crosslinker, while the surface ions coordinate with chitosan (CS) to form a second network, resulting in NICH. This hydrogel was coated onto ZnO nanowires for bacterial detection. When bacterial samples are dropped onto this platform, the synergistic bactericidal effects of the nanowires (mechanical piercing) and the hydrogel (enhanced adhesion) allow for effective bacterial capture and inactivation. Second, upon bacterial lysis, the released glutathione (GSH) reduces the oxidized form of TMB (TMB ox), resulting in color change that can be quantified through colorimetric analysis. Lastly, the color change of the entire reaction is detected by the smartphone to determine the bacterial concentration.

In the lungs of mammals, mucociliary clearance plays a key role in not only protecting tissues but also the capturing and sensing of inhaled foreign pathogens.^[^ ^20^ ^]^ The thin mucus gel^[^ ^21^ ^]^ infuses and covers the underneath periciliary layer, containing encapsulated polysaccharides and glycoproteins, such as bacteria‐killing enzymes, which can disinfect large pathogens.^[^ ^22^ ^]^ The dense hairy cilia can prevent the invasion of submicron pathogens and keep them retained in the mucus gel for further clearance. Inspired by pulmonary cilia and the mucus on them, we propose a nanozyme‐centric hydrogel, specifically, nanozymes (Cu₂O) – initiated and crosslinked hydrogel (NICH), which is assembled onto ZnO nanowires for integrated bacterial adhesion, lysis, and detection (**Figure** **1**). Notably, NICH mimics the functions of respiratory mucus,^[^ ^23^ ^]^ with adhesive properties that facilitate the rapid capture of pathogens. In contrast, nanowires are regarded as rigid cilia designed to penetrate pathogens. Specifically, we established the foundational gel framework by utilizing Cu₂O nanozymes to catalyze the polymerization of acrylamide monomers (AAM). The addition of chitosan (CS) promotes the formation of coordination bonds with Cu₂O nanoparticles, thereby enabling the uniform encapsulation of the nanozymes. The resultant nanozyme‐centric hydrogel can be assembled onto ZnO nanowires in a controlled manner, creating a hydrogel adhesive layer that facilitates the rapid capture of bacteria. Moreover, the inherent hydrophilicity and water‐absorbing properties of the hydrogel layer enable the effective absorption and confinement of lysates, facilitating direct interaction with the uniformly encapsulated nanozymes for colorimetric bacterial detection. By integrating the mechanical piercing of nanowires with the adhesive capture and retention of lysate of hydrogels, we achieved efficient in situ bacterial lysis while markedly reducing the risk of infection. Given the straightforward fabrication of nanowires and the controllable assembly of the transparent hydrogel, our platform demonstrates high adaptability to various substrates. Ultimately, the incorporation of mobile signal recognition offers significant promise for rapid home testing in resource‐limited environments.

## 2 Results and Discussion

### 2.1 The preparation of Nanozymes (Cu₂O) – Initiated and Crosslinked Hydrogel (NICH)

**Figure 2:**
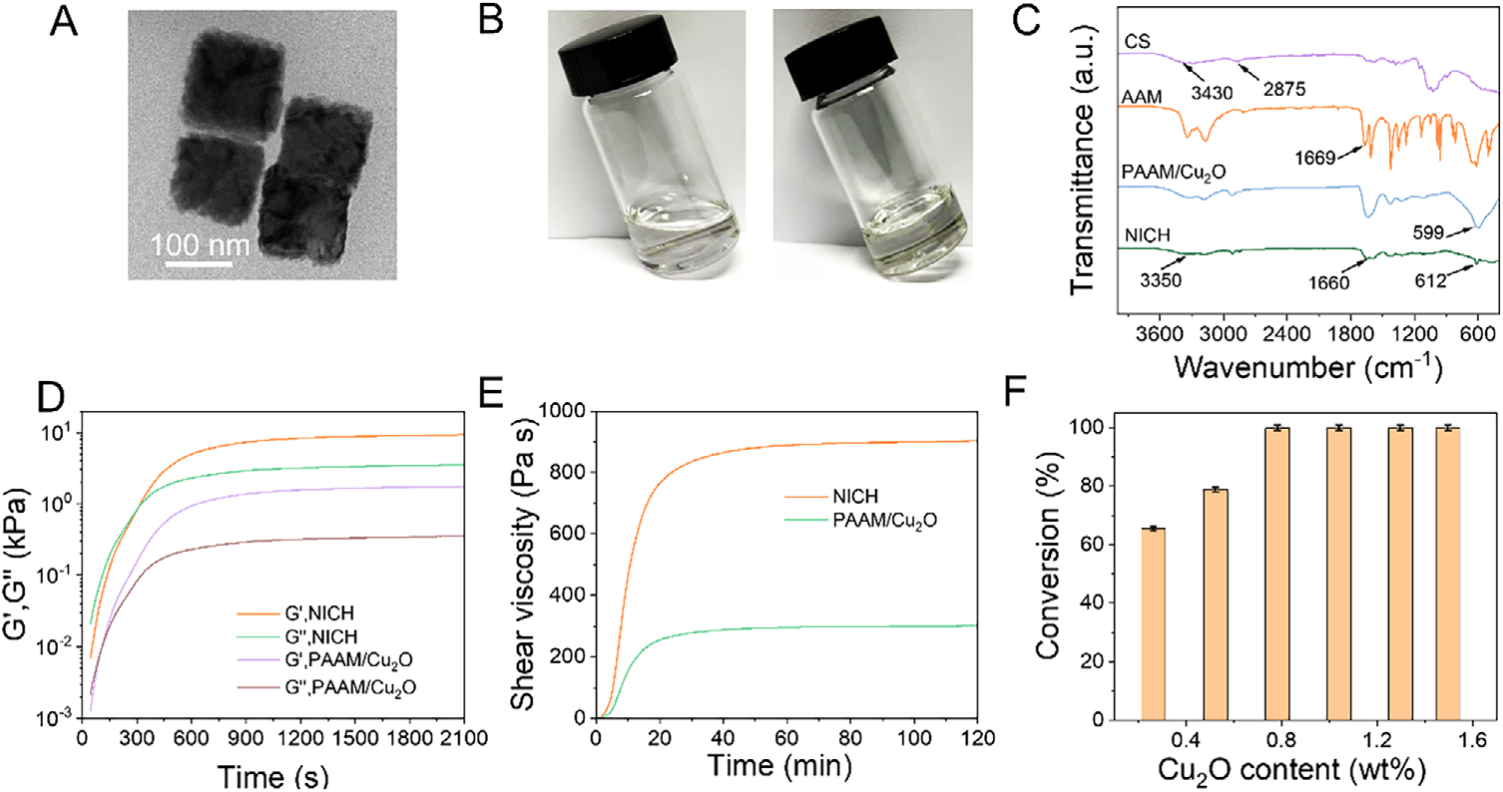
The formation of nanozymes (Cu₂O) – initiated and crosslinked hydrogel (NICH). A) The TEM image of Cu_2_O nanoparticles. B) Optical photos before (left) and after (right) hydrogel formation. C) Fourier‐transform infrared (FTIR) spectroscopy of NICH. D) Dynamic time sweeps of NICH and PAAM/Cu_2_O hydrogel at a frequency of 1 Hz. E) Time‐dependent viscosity changes in NICH and PAAM/Cu_2_O hydrogel. F) Relationship between monomer conversion and content of Cu_2_O nanoparticles. The reaction time was 40 min, n = 3, mean ± SD.

First, Cu₂O nanoparticles were synthesized using a chemical reduction method (for detailed procedures, see the supplementary materials), yielding cube nanoparticles with the side length of 100 nm (**Figure** **2**A). X‐ray photoelectron spectroscopy (XPS) analysis of Cu₂O nanoparticles revealed a Cu 2p₃/₂ signal at 932 eV, indicating the presence of Cu⁺ oxidation state. A Cu 2p₁/₂ signal at 952 eV and an O 1s peak at 932 eV further confirm the successful synthesis of the material (Figure S1, Supporting information). Afterward, we conducted a systematic investigation into the formation of Cu_2_O nanozymes‐centric hydrogel. It is evident from the photographs in Figure 2B that the liquid (left) forms a hydrogel (right) after adding Cu_2_O nanoparticles. Further characterization by Fourier‐transform infrared (FTIR) spectroscopy provides insights into the chemical interactions within the hydrogel. As shown in Figure 2C, the FTIR spectrum of NICH exhibits characteristic peaks for CS, including the O─H and N─H stretching vibrations ≈3430 cm^−1^, the C─H stretching vibrations at 2875 cm^−1^. After free radical polymerization, the C═C vibration peak at 1620 cm⁻¹ related to the vinyl group became unidentifiable. The C═O stretching vibration peak of the amide group shifts from 1669 cm⁻¹ (AAM) to 1660 cm⁻¹ (NICH) due to hydrogen bonding with the ‐NH₂ or ─OH groups of CS. Additionally, the O‐H stretching vibration of CS at 3430 cm⁻¹ shifts to 3350 cm⁻¹ after NICH forms, likely attributed to hydrogen bonding with polyacrylamide (PAAM). The appearance of the peak at 624 cm^−1^ corresponds to the Cu (I)‐O vibrations, further confirming the incorporation of the Cu_2_O nanoparticles into the hydrogel network. The formation of NICH is further evidenced by the X‐ray diffraction (XRD) shown in Figure S2 (Supporting information), which reveals the characteristic diffraction peaks corresponding to the (110), (111), (220), (310), and (321) crystal planes of Cu_2_O nanoparticles, indicating the successful integration of the Cu_2_O nanoparticles into the hydrogel structure.

To elucidate the formation of the hydrogel and examine its behavior upon the introduction of chitosan (CS), we conducted an extended monitoring of the dynamic gelation process. As illustrated in Figure 2D, the gel point for the system devoid of CS is observed at ≈100 s, whereas for the CS‐containing system occurs ≈300 s. This disparity may be attributed to the simpler gelation mechanism in the former. Notably, both two systems exhibit rapid gelation processes, clearly demonstrating that the incorporation of CS significantly enhances the network. The viscosity of the hydrogel stabilizes after ≈40 min (Figure 2E), thereby confirming the successful synthesis of the hydrogel. Crucially, the amount of Cu_2_O nanoparticles used as the crosslinker was found to play a crucial role in hydrogel formation. The conversion of hydrogels was tested by the freeze‐drying weighing method. As depicted in Figure 2F, when the content of Cu_2_O nanoparticle reached 0.78 wt%, the monomer conversion rate reached 99.9%, indicating the high efficiency of the nanozyme‐triggered polymerization.

### 2.2 Mechanism and Optimization of Hydrogels Prepared by Cu_2_O Nanoparticles as Enzymatic crosslinkers

**Figure 3:**
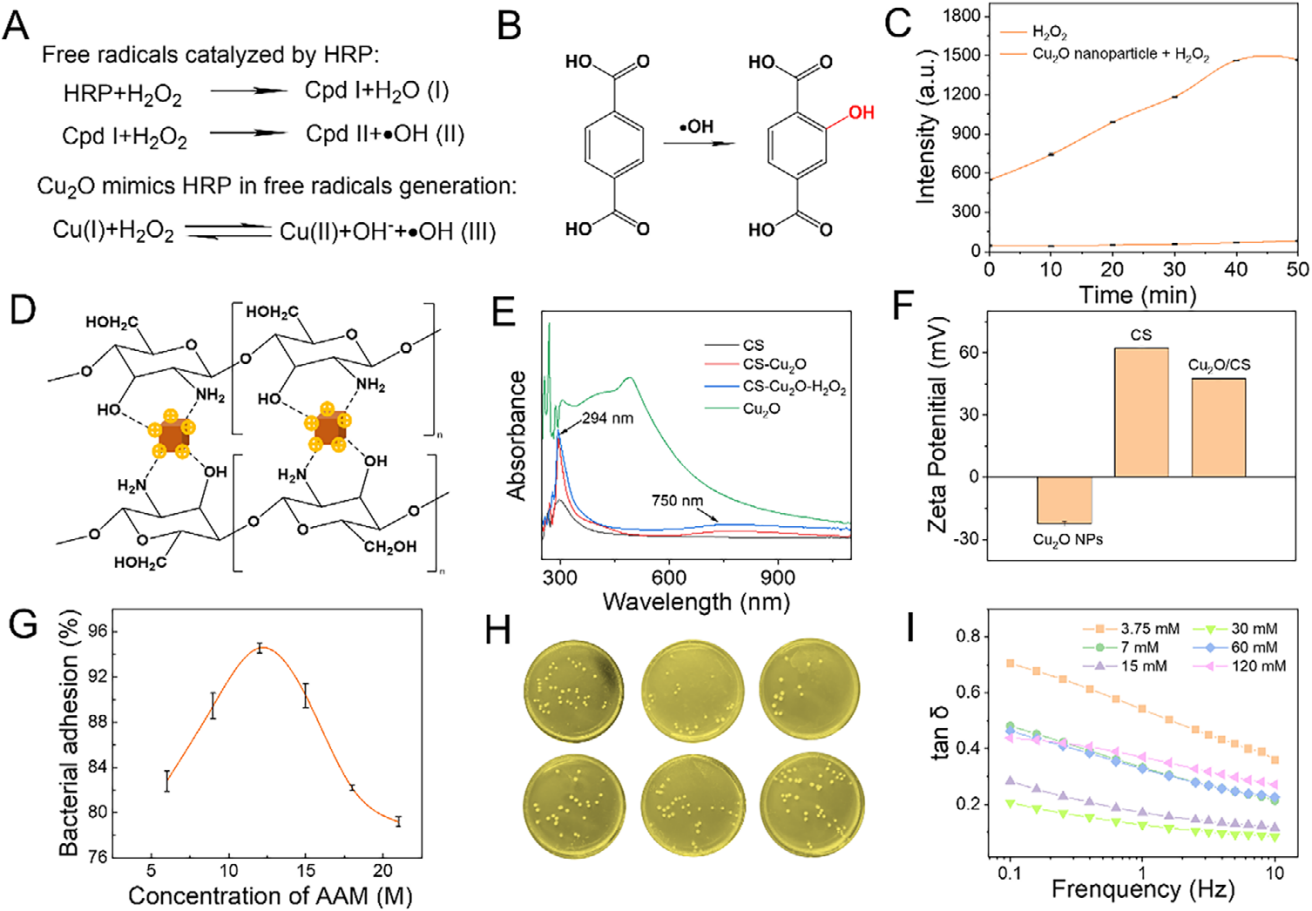
Mechanism and optimization of hydrogels prepared by Cu_2_O nanoparticles as enzymatic crosslinkers. A) (I) (II) Reaction equation for generating free radicals catalyzed by HRP; (III) The reaction equation for Cu₂O nanozyme mimics the free radical generation process of HRP. B) The reaction of terephthalic acid to capture hydroxyl radicals. C) Comparison of fluorescence intensity of o‐hydroxybenzoic acid produced by trapping free radicals, n = 3, mean ± SD. D) Schematic diagram of copper ion coordination on the surface of CS and Cu_2_O nanoparticles. E) UV‐visible spectroscopy (UV–vis) spectrogram of CS/Cu_2_O. F) Electrostatic interactions between CS and Cu_2_O nanoparticles, n = 3, mean ± SD. G) Bacterial adhesion rates and photos of bacterial cultures H) to NICH formed from different concentrations of AAM, n = 3, mean ± SD. (I) Loss factor (tan δ) as a function of frequency for NICH with varying concentrations of CS.

Analogous to the reaction principles illustrated in **Figure** **3**A (I) and (II), where HRP catalyzes the generation of hydroxyl radicals, Cu₂O nanoparticles demonstrate mimetic peroxidase activity, positioning them as an ideal substitute for HRP (Figure 3A). We verified the peroxidase activity of Cu_2_O nanoparticles by measuring their kinetic parameters (Figure S3, Supporting information). Interestingly, the Cu_2_O nanoparticles exhibit a higher maximum velocity (V_max_) and lower Michaelis constant (K_m_) compared to the HRP enzyme (Figure S3E), suggesting superior catalytic efficiency and substrate affinity. For the follow‐up detection, we further explored whether encapsulation would impact the catalytic activity of the nanozymes. It is observed that the activity of the nanozymes encapsulated in the hydrogel remained relatively stable, which is highly advantageous for colorimetric detection (Figures S3D,E, Supporting information). To demonstrate the production of hydroxyl radicals during the catalytic process, we trapped the radicals by terephthalic acid (Figure 3B). The results showed that the Cu_2_O nanoparticles can catalyze the generation of free radicals (Figure 3C), which provides the foundation for the polymerization of AAM monomers triggered by the free radicals. Furthermore, the CS in the system is immobilized by forming coordination bonds with the free Cu^2+^ and Cu^+^ on the surface of the Cu_2_O nanoparticles (Figure 3D). The UV–vis spectrum further confirmed the coordination bonds between Cu^2^⁺ (750 nm), Cu⁺ (294 nm), and CS^[^ ^24^ ^]^ (Figure 3E). At the same time, the zeta potential of the Cu₂O nanoparticle is −22 mV, while that of CS is + 67 mV. The electrostatic attraction between them leads to partial neutralization upon combination, resulting in a composite zeta potential of + 48 mV (Figure 3F). This enhances the stability of the composite, which retains a positive charge, facilitating the capture of negatively charged bacteria.

The assembly of NICH on the nanowire platform for lysis and detection involves two critical factors: the stability and adhesiveness of NICH. Initially, we examined the influence of adhesion on bacterial attachment by fabricating hydrogels with varying concentrations of AAM. We observed that as the AAM concentration increased, bacterial adhesion exhibited a trend of initial enhancement followed by a decline (Figures 3G,H). We think that at lower concentrations, the hydrogel network remains relatively porous, impeding bacterial adhesion. Conversely, at excessively high concentrations, the augmented mechanical strength of the hydrogel compromises its adhesiveness. Consequently, we identified 12 M AAM as the optimal condition for our experiments. While the adhesive properties of NICH are integral to bacterial lysis, the stability is equally critical for the bactericidal effect on the nanowires. Our findings indicated that as the concentration of chitosan (CS) increased, the loss factor (tan δ) initially decreased before subsequently rising (Figure 3I). This behavior can be attributed to the difficulties in forming a dense hydrogel network at lower CS concentrations. As the CS concentration increases, the degree of crosslinking strengthens, ultimately reaching optimal crosslinking at 30 mM of CS. However, at excessively high CS concentrations, the resultant overabundance of interactions may induce brittleness in the network, thereby diminishing its energy absorption capacity and leading to an increase in tan δ. Moreover, shear viscosity assessments demonstrated that at the concentration of 30 mM, not only was the crosslinking degree optimal, thus ensuring stability, but it also exhibited relatively high viscosity, which is advantageous for promoting subsequent bacterial adhesion (Figure S4A, Supporting information). Moreover, as illustrated in Figure S4B (Supporting information), the incorporation of chitosan (CS) leads to superior storage and loss moduli of NICH compared to the PAAM/Cu_2_O hydrogel. This enhancement likely pertains to an increased crosslinking density within the hydrogel, thereby reinforcing our earlier conclusions.

### 2.3 ZnO Nanowires and Their Long‐Term Antibacterial Application in Melt‐Blown Fabrics

**Figure 4:**
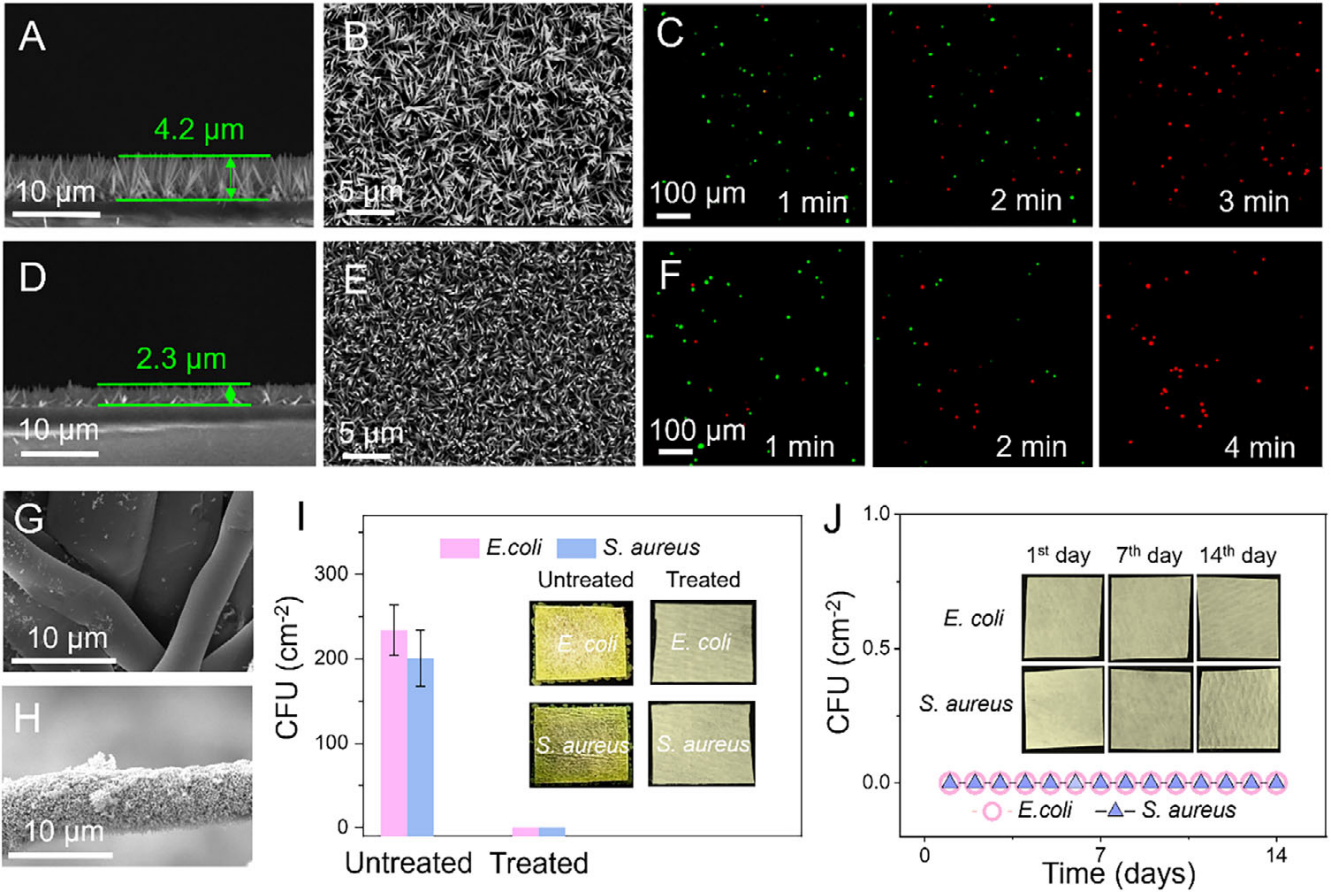
ZnO nanowires and their long‐term antimicrobial applications. SEM images: side view A) and top view B) of 4.2 µm nanowires. C) Fluorescent sterilization images of 1 µL of *E. coli* using 4.2 µm ZnO nanowires. SEM images: side view D) and top view E) of 2.3 µm nanowires. F) Fluorescent sterilization images of 1 µL of *E. coli* using 2.3 µm ZnO nanowires. G) SEM images of melt‐blown fabric and H) ZnO nanowire array‐decorated melt‐blown fabric. I) The colony forming units of *E. coli* and *S. aureus* are 234 VS 0 cm^2^ and 201 VS 0 cm^2^, n = 3, mean ± SD. The inset pictures show corresponding samples (2 × 2 cm). (J) Colony formation assay to demonstrate excellent antibacterial performance for two weeks. No colony unit is found as indicated in inset photographs.

ZnO nanowires function as substrates for NICH and as primary agents for penetrating bacteria, rendering their antibacterial properties critical. Initially, we synthesized ZnO nanowires of different lengths with sharp nanostructures (**Figure** **4**B,E), specifically measuring 4.2 µm (Figure 4A) and 2.3 µm (Figure 4D). We subsequently assessed the bactericidal efficacy of both types of nanowires against *E. coli*, revealing that the 4.2 µm nanowires (Figure 4C,F) demonstrated superior antibacterial activity, which confers enhanced capillarity and wicking capabilities. Amid the swift proliferation of respiratory infectious diseases worldwide, we explored the integration of ZnO nanowires into melt‐blown fabric for aerosol disinfection. Our observations indicated that the melt‐blown fabric containing the nanowires became noticeably rougher, with densely packed nanowires clearly visible (Figure 4G,H). For antibacterial applications on melt‐blown fabric or similar surfaces, long‐term efficacy is of utmost importance. We found that the melt‐blown fabric with incorporated nanowires not only retained antibacterial properties but also maintained their effectiveness for up to 14 days (Figure 4I,J). This has profound implications for the sustained antibacterial performance of masks and various surfaces, potentially reducing the frequency of equipment surface replacements.

### 2.4 Bactericidal Performance of the Platform Prepared by NICH Assembled onto Nanowires

**Figure 5:**
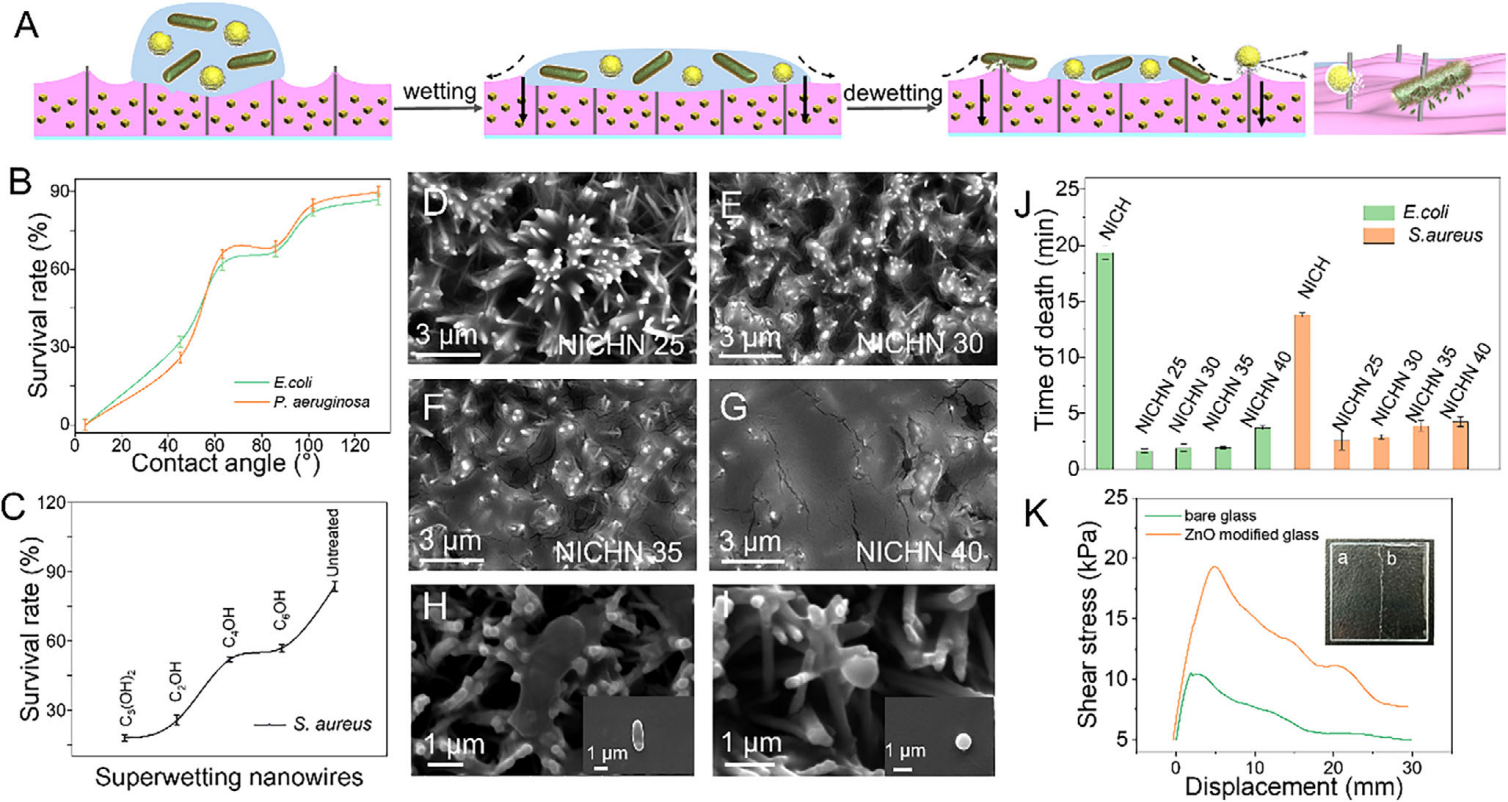
Bactericidal performance of the platform prepared by NICH assembled onto nanowires. A) The process of spreading and lysis of bacteria on the platform prepared by NICH integrated into nanowires. B) Dependence of *E. coli* and *P. aeruginosa* survival rate on the wettability of nanowire surfaces characterized by the water contact angle. Hydrophilic surfaces result in reduced survival rates, n = 3, mean ± SD. C) Survival rates of droplet‐carried *S. aureus* on superwetting ZnO nanowires, n = 3, mean ± SD. The hydrogels formed by different contents of gel precursors were loaded into nanowires, with precursors of D) 25 µL named as NICHN 25, E) 30 µL named as NICHN 30, (F) 35 µL named as NICHN 35, and G) 40 µL named as NICHN 40, respectively. SEM images of NICHN 25 for H) *E. coli* and (I) *S. aureus* lysis. J) The killing time of different materials against *E. coli* (green) and *S. aureus* (orange), n = 3, mean ± SD. K) Comparison of shear strength of hydrogels coated on smooth glass and on glass slides with ZnO nanowire arrays. The image (a) shows bare glass (left), while image (b) displays glass coated with the hydrogel (right).

As shown in **Figure** **5**A, we demonstrate the rapid wetting and dewetting processes of droplets on a superhydrophilic substrate. Subsequently, the combination of the adhesion properties of the hydrogel and the capillary action of the nanowires significantly enhances the downward spreading tendency of the bacteria. After bacterial lysis, the resulting lysate is absorbed and confined within the hydrogel. Having proved the bactericidal efficacy of the nanowires, we now proceed to modify them to facilitate the lysis of bacteria with diverse morphologies. To ensure safe detection in the future, we first focus on enhancing bactericidal efficiency. By adjusting the ratio of HSC₆OH to HSC₂ + HSC₆OH (φ), we found that as φ increased from 20% to 100%, the contact angle decreased (Figure S5A, Supporting information), indicating enhanced hydrophilicity, which also improved the antibacterial effect. When the nanowires achieve a superhydrophilic state (φ = 100%), the bactericidal rates against *Escherichia coli* (*E. coli*) (Figure S5DFigure S6A, Supporting information) and *Pseudomonas aeruginosa* (*P. aeruginosa*) (Figure S5E, Supporting information) reach 100% (Figure 5B). Intriguingly, the maximum bactericidal efficiency against *Staphylococcus aureus* (*S. aureus*) is 83%, significantly lower than that observed for *E. coli*, even after hydrophilic modification (Figure 5C; Figures S5B and S6B, Supporting information). This difference may be attributed to the relatively small contact area of *S. aureus* and the insufficient spreading caused by its aggregation.

To further design a solution for eliminating spherical bacteria and enhancing bactericidal efficiency against rod‐shaped bacteria, we combined hydrogels (Figure S7, Supporting information) with nanowires. The elemental analysis confirms the successful and uniform encapsulation and dispersion of Cu_2_O nanoparticles (Figure S7B, Supporting information). Moreover, the results of Particle Dispersion Index (PDI) further indicate that, compared to bare nanoparticles, the PDI of encapsulated nanoparticles decreases, suggesting that they are less prone to aggregation (Figure S8, Supporting information). The picture and structure of the NICH assembled onto the nanowires is depicted in Figure S9 and S10 (Supporting information). For investigating the enhanced lysis, NICH of varying volumes (25 µL, 30 µL, 35 µL, and 40 µL) were assembled onto the nanowires (NICHN) (Figure S9, Supporting information), labeled as NICHN 25 (Figure 5D), NICHN 30 (Figure 5E), NICHN 35 (Figure 5F), and NICHN 40 (Figure 5G), respectively. We found that standalone nanowires achieved a bactericidal time of ≈3.7 min against *E. coli*, demonstrating significant deformation, consistent with prior plate experiments. In contrast, standalone NICH required 19.3 and 13.8 min to eliminate *E. coli* and *S. aureus*, respectively. Notably, NICHN 25 exhibited the highest bactericidal efficiency (Figure 5J), with killing times of 1.6 min for *E. coli* (Figure S11, Supporting information) and 2.6 min for *S. aureus* (Figure S12, Supporting information). Following treatment with NICHN 25, both *E. coli* (Figure 5H) and *S. aureus* (Figure 5I) displayed morphological changes and membrane folding, indicating robust antibacterial activity. Compared to pure hydrogel, NICHN 25 demonstrated superior and faster bactericidal effects. This enhancement may arise from the optimal quantity of hydrogel precursor, which promotes bacterial adhesion without fully obscuring the nanowires. This balance not only preserves the puncturing effect of the nanostructures but also amplifies it through the adhesive properties of NICH. Conversely, NICHN 40 showed the least effective bactericidal performance, possibly due to an overly thick NICH coating, where the antibacterial effect was mainly driven by electrostatic interactions of CS in the hydrogel, leading to prolonged killing times. Crucially, the combined platform outperformed individual components, underscoring the synergistic bactericidal effect achieved through the integration of nanowires and hydrogel. Simultaneously, to investigate potential influences on the sterilization effect from other factors, we examined the impact of ions released from two materials. The results indicate that the ion release is minimal, exerting negligible effects on bacterial cultures. (Figure S13, Supporting information). Considering these findings, we have opted to proceed with NICHN 25 for further detection studies.

To further demonstrate characteristics in practical applications, we coated the hydrogel into nanowire arrays and compared it to a bare glass substrate. The results indicate that the shear strength is larger when the hydrogel is integrated into the nanowire array than that of bare glass (Figure 5K). This phenomenon can be attributed to the confinement effect of the nanowires on the hydrogel, which may be beneficial for the stability of the system and subsequent bacterial adhesion. Furthermore, the inset images reveal that the hydrogel demonstrates exceptional transparency, with virtually no discernible difference between the left (a) and right (b) sides of the glass slide. This characteristic establishes a robust foundation for colorimetric detection. By optimizing the hydrogel composition and understanding its rheological behavior on nanowires, we have laid the groundwork for the effective applications of these bioinspired materials in targeted biomedical settings.

### 2.5 Demonstration of Bacterial Sensing on the Integrated Platform

**Figure 6:**
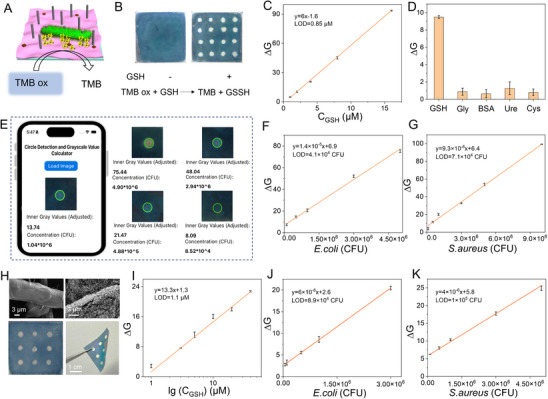
Quantitative analysis of bacteria based on integrated methods. A) Schematic illustration of the integrated platform and GSH biosensing using a developed smartphone application. B) Demonstration of colorimetric detection array by adding GSH to the glass slide‐based detection platform, the color of the coated area noticeably lightens. C) The bacterial detection platform is utilized to analyze GSH standard solutions to obtain a standard curve. D) Specific detection is conducted by incorporating various interfering substances, including glycine (Gly), bovine serum albumin (BSA), urea (Ure), and cysteine (Cys). E) Interface illustration of the bacterial detection application on a mobile app, presenting the results for *E. coli* at varying concentrations. F) Linear correlation between the concentration of *E. coli* and the corresponding grayscale value. G) Relationship between the concentration of *S. aureus* and grayscale values. H) SEM images of A4 paper and A4 paper coated with hydrogel and nanowires, as well as front views and bent optical photos of a bacterial detection platform based on 2 cm × 2 cm paper. I) Calibration curve obtained for GSH using a detection platform made of A4 paper. The paper‐based detection platforms are used for detecting *E. coli* (J) and *S. aureus* K). The error bars represent the standard deviation (n = 3).

The level of glutathione (GSH) in the body serves as a strong biomarker for various diseases, including differentiation of cancer cells^[^ ^25^ ^]^ and bacterial infections.^[^ ^26^ ^]^ We utilized Cu₂O nanozymes encapsulated in hydrogels for signal transduction amplification to develop the colorimetric assay for detecting glutathione (GSH) leakage from bacteria (**Figure** **6**A). The oxidation product of 3,3′,5,5′‐tetramethylbenzidine (TMB ox) is reduced by GSH, resulting in a lighter blue color (Figure 6B). Through comparative analysis, we determined that the residual free radicals within the hydrogel and its intrinsic components did not exert a significant influence on the colorimetric reaction (Figure S14, Supporting information). To achieve self‐testing of bacteria, we developed a smartphone application (Figure 6E) that can quickly identify changes in gray values to calculate bacterial concentration. To facilitate the interaction between the hydrogel and the GSH produced by bacterial lysis, the hydrogel network was loosened by treating it with a 1 M NaCl solution for ≈3 min after the bacterial droplets were sprayed on the integrated platform (Figure S15, Supporting information). The detection platform was tested with 1–64 µM of GSH, and a linear relationship was observed between 1–15 µM, with a limit of detection (LOD) of 0.85 µM (Figure 6C). To further verify the specificity, interfering molecules, including cysteine (Cys), bovine serum albumin (BSA), glycine (Gly), and urea (Ure) were evaluated (Figure 6D). Formaldehyde was used as a sulfhydryl masking agent to improve the selectivity of GSH determination.^[^ ^27^ ^]^ The integrated detection platform was then used to detect *E. coli* and *S. aureus* with the LOD of 4.1 × 10^4^ CFU and 7.1 × 10^4^ CFU respectively. Moreover, we found that the platform exhibited excellent reproducibility (Figure S16, Supporting information). In comparison with recent studies, our method exhibits a rapid response without the requirement for large‐scale instruments or antibodies. Additionally, it ensures safety by eliminating bacteria prior to detection (Table S1, Supporting information). To further reduce the cost of the detection platform, we explored the use of portable paper as substrates. As illustrated in Figure 6H, after modifying the paper with nanowires and hydrogel, the previously smooth structure becomes noticeably rougher, and the dimensions of the paper are 2 cm × 2 cm. Additionally, we validated the detection of glutathione (GSH) (Figure 6I) and assessed its LOD for *E. coli* and *S. aureus*, which were found to be 8.9 × 10⁴ CFU (Figure 6J) and 1 × 10⁵ CFU (Figure 6K), respectively. Notably, in comparison to glass‐based detection platforms, there is a modest decline in sensitivity, which may be attributed to the greater irregularity of the paper substrate. However, according to reported work, bacterial infection can be identified when the bacterial concentration exceeds 10^5^ CFU. Therefore, this platform can provide a safe method for patient self‐testing via mobile devices and hold promises for on‐site bacterial testing in resource‐limited settings.

It is important to note that our design possesses versatility because the application of nanostructures^[^ ^28^ ^]^ for bacterial penetration can extend beyond the ZnO nanowires to encompass silicon nanowires as well. Silicon nanowires are recognized for several distinct advantages: first, silicon demonstrates exceptional electrical conductivity,^[^ ^29^ ^]^ which enhances the signal sensitivity of biosensors. Second, the surface functionalization techniques applicable to silicon nanowires allow for precise tuning, thereby improving the selective adsorption of specific bacteria. Lastly, silicon materials can be patterned using masking techniques, simplifying the design of microfluidic chips and facilitating seamless integration with other microelectronic components to create sophisticated sensor systems.^[^ ^30^ ^]^ As illustrated in Figure S17 (Supporting information), the silicon nanowires we fabricated measure ≈8.7 ± 0.6 micrometers. We scraped the silicon nanowires for FTIR testing, revealing a peak at 623 cm⁻¹ for the Si‐Si bond and a peak at 1065 cm⁻¹ for the Si─O─Si bond stretching vibration (Figure S18, Supporting information). Through bacterial staining, we observed complete eradication of bacteria following treatment with silicon nanowires, underscoring their effective bactericidal properties. As we mentioned, we also observed the wetting and dewetting processes on the hydrophilic silicon nanowires (Figure S19, Supporting information). To further investigate the impact of hydrophilicity on bactericidal efficacy, we modified the silicon nanowires and found that a reduction in contact angle correlated with enhanced bactericidal effects against *E. coli* and *P. aeruginosa* (Figure S20, Supporting information). The SEM images reveal pronounced alterations in the silicon nanowire before and after the application of the NICH (Figure S21, Supporting information). Thus, silicon nanowires can serve as an alternative to the ZnO nanowires used in our original study, demonstrating their potential for the scalable fabrication of advanced devices.

## 3 Conclusion

We developed nanozymes (Cu₂O) – initiated and crosslinked hydrogel (NICH) and assembled it onto ZnO nanowires for enhanced lysis, with the nanozymes uniformly encapsulated for colorimetric detection. First, we managed to use Cu_2_O nanoparticles as both the initiator and the crosslinker to prepare nanozyme‐centric hydrogel, without using any hazardous substances. It is important to emphasize that, given that other nanozymes may also possess the capability to initiate hydrogel formation, we can substitute the Cu_2_O nanoparticles used in this study with alternative nanozymes. This approach provides a new perspective on the problem of uneven encapsulation of nanozymes. Second, ZnO nanowires can serve as a long‐lasting antibacterial surface, and the assembly of hydrogels onto the nanowires further enhances their bactericidal efficacy. By tuning the content of hydrogel precursor, we were able to optimize the bactericidal performance of the nanowires. The preparation and assembly of our nanozymes and hydrogels are remarkably straightforward, rendering them compatible with versatile substrates. Furthermore, the integration of machine learning significantly amplifies their potential in resource‐constrained environments and for self‐diagnosis.

## 4 Experimental Section

### Preparation for ZnO Nanowires

First, ZnO seeds was synthesized according to the following method.^[^ ^31^ ^]^ Dissolve 1.10 g (5.0 mmol) of zinc acetate dihydrate in 50.0 mL of isopropanol to prepare a 100 mM solution. Then, vigorously stir the resulting solution at 85 °C for 15 min. After that, gradually add 700 µL of triethylamine (5.0 mmol) dropwise to the stirring solution. Allow the clarified solution to stand for 3 hours to cool for later use. This was revised the methodology of the ZnO nanowires.^[^ ^32^ ^]^ Apply the seed solution onto the substrate, which can be replaced with the corresponding material, such as melt‐blown fabric or paper, and after curing at 105 °C for half an hour, prepare the growth solution using 15 mM hexamethylenetetramine (HMTA) and 15 mM zinc nitrate hexahydrate (Zn(NO_3_)_2_·6H_2_O) as precursors, adding 0.8 M ammonia to promote the growth at 95 °C for 6 hours.

### Fabrication of Silicon Nanowires

Silicon nanowires were made by etching ⟨100⟩ n‐type silicon wafers. First, the wafers were treated with a Piranha solution of H₂SO₄ and H₂O₂ (3:1) for 10 min. They were then immersed in a catalyst solution of 4.8 M hydrofluoric acid and 0.01 M silver nitrate for 1 min. Next, the wafers were etched in a solution of 4.8 M HF and 0.3 M H₂O₂ for 21 min. After etching, the wafers were placed in dilute nitric acid to remove silver catalysts and were washed with deionized water before air drying.

### Preparation of Cu_2_O Nanoparticles

Cu_2_O nanoparticles was synthesized according to the literature.^[^ ^33^ ^]^ Typically, an aqueous solution of CuCl_2_ (33 mL, 3.20 mM) is added to a 50 mL flask. Then, under magnetic stirring, NaOH aqueous solution (1 mL, 0.35 M) was added, and the reaction solution slowly changed from colorless to light blue. After 5 min, sodium L‐ascorbate aqueous solution (1 mL, 0.10 M) was rapidly injected into the above solution and maintained for 30 min. The resulting Cu_2_O nanoparticles were collected by centrifugation and washing with deionized water/ethanol.

### Bacteria Culture

Gram‐positive spherical bacteria *Staphylococci aureus* (*S. aureus*), gram‐negative rod bacteria *Pseudomonas aeruginosa* (*P. aeruginosa*), and *Escherichia. Coli* (*E. coli*) were cultured to prepare the corresponding bacteria solution. A single colony of the bacteria on a solid Luria−Bertani agar plate was transferred in 10 mL Luria−Bertani broth for further culture of 16 hours at 37 °C. The bacteria solution was washed with PBS (Invitrogen) and collected with centrifugation at 7000 g (Sigma, 3‐15–3k15) for 10 min. The bacteria pellet was resuspended with PBS, and the suspension was ready for further experiment. Simultaneously, the bacteria concentration was measured by serial dilution and the colony‐forming units counting.

### Live/Dead Fluorescence Characterization of Bacteria

1 mL bacteria suspension of 10^5^ CFU/mL was stained with 1 µL SYTO 9 and 1 µL PI for 20 min at room temperature. After incubation, the stained bacteria solution was centrifuged at 7000 g for 10 min to remove the supernatant. Then, 800 µL PBS was added for resuspending bacteria. The fluorescence was observed and recorded with fluorescence microscopy (Eclipse Ni‐E). The green fluorescence SYTO 9 was recorded at 503 nm with the excitation of 488 nm. The red fluorescence PI was recorded at 620 nm with the excitation of 532 nm. Bacteria with intact cell membranes were stained with fluorescent green, whereas bacteria with damaged membranes were stained with fluorescent red.

### Preparation of Nanozyme Initiated Hydrogels

Hydrogels were prepared by free radical polymerization of Cu_2_O nanoparticles. In general, the solution of chitosan with a concentration of 0.5 wt% was prepared by dissolving chitosan in acetic acid. AAM (0.03 mol), MBAA (0.0108 mol% of AAm) 10 µL 30 wt% H_2_O_2_, and nanozyme (200 µL, 57 mg/ mL^−1^) were added to 2.5 mL CS solution with stand for 1.5 hours.

### Mechanism of Nanozyme Initiated Hydrogel

OH was detected by fluorescence method with terephthalic acid as a probe molecule. Terephthalic acid (0.5 mM), sodium hydroxide (2 mM) and catalyst (0.5 mg mL^−1^) were dispersed in 10 mL aqueous solution. The fluorescence spectra of the generated 2‐hydroxyterephthalic acid were measured by a Hitachi F‐7000 fluorescence spectrophotometer excited at 315 nm.

### Mechanical Properties of Hydrogels

The rheological properties of hydrogel were tested by a rheometer. The hydrogel samples were placed between parallel plates 10 mm in diameter and 1 mm apart. The elastic modulus (G’) and viscosity modulus (G’’) are measured at 25 °C at a frequency sweep of 0.1–10 Hz, with a strain of 1%.

### Preparation of the Integrated Bacterial Detection Platform

First, the square glass sheet with a side length of 25 mm was ultrasonic cleaned, and the zinc oxide seed drops were coated on the glass plate. After heating and curing at 105 °C for 30 min, the growth solution was prepared with 15 mM hexamethylenetetramine and 15 mM zinc nitrate hexahydrate as the precursor system, and 0.8 M ammonia water was added, and the growth was kept at 95 °C for 6 h. Then, the precursor solution (25–40 µL) was applied to the glass plate, sealed and stored, and triggered in situ for 40 min to obtain a portable detection platform.

### GSH Biosensing by Prepared Platform

20 mg mL^−1^ TMB and 10 mM H₂O₂ solution were added to the test platform for incubation, followed by the addition of different concentrations of GSH (1–64 µM). After incubating for 15 min in the dark, the Nikon D5500 camera was used to capture the RGB color, which was then loaded into Image J software or the Apps this was made to obtain gray values.

### Bacteria Biosensing by Prepared Platform

20 mg mL^−1^ TMB and 10 mM H₂O₂ solution were added to the testing platform (pH 4.0) for incubation. Then, formaldehyde was added to block other interfering substances, and NaCl was added to make the hydrogel loose. Different concentrations of *E. coli* and *S. aureus* (5×10⁴–5×10⁷ CFU) were then dropped onto the platform. After incubating for 15 min in the dark, the Nikon D5500 camera was used to capture the RGB color, which was then loaded into ImageJ software or the Apps this was made to obtain gray value.

### Statistical Analysis

Each experiment was performed in triplicate and results are expressed as mean ± standard deviation (n = 3). Statistical analysis and related graphs were generated using Origin 2023 software and its integrated functions. The detection section is represented by experimental data and fitting curves.

## Conflict of Interest

The authors declare no conflict of interest.

## Author Contributions

X. Y. supervised the project. X. Y., and Z.T. planned the study. Z.T. performed the experiments with support from X. W., J.J. and Z.W. for general characterizations. Z.W. assisted with optical photographs, and Z.W. and X. W. assisted with practical application demonstrations. Z.T. wrote the original manuscript. All the authors discussed the analysis of the results and commented on the manuscript. This work was supported by the Research Grant Council of Hong Kong (CityU 11 307 220 and CityU 11 307 721), Shenzhen Basic Research Program (JCYJ20240813153107010), Guangdong Basic Research Program (2025A1515011479), and IDM Project (9229501‐14‐YX) from City University of Hong Kong.

## Supporting information



Supporting Information

## Data Availability

The data that support the findings of this study are available in the supplementary material of this article.
