# Supplementary material for: Mucociliary Clearance Inspired Nanozyme‐centric Hydrogel Composites for Integrated Bacterial Detection
Source: Adv Sci (Weinh). 2025 Jul 21;12(36):e03809. doi: 10.1002/advs.202503809 (PMC12463100; doi:10.1002/advs.202503809)
Supplement: Supplementary file 1 — Supporting Information [file ADVS-12-e03809-s001.docx]

**Supporting Information**

**Mucociliary Clearance Inspired Nanozyme-centric Hydrogel Composites for Integrated Bacterial Detection**

Zhaoling Tan^1^, Xuejiao Wang^1^, Zhaoyue Wang^3^, Jieke Jiang^4^, Xi Yao*^1,2^

1 Department of Biomedical Sciences, City University of Hong Kong, Kowloon, Hong Kong SAR 999077, P. R. China.

2 Shenzhen Research Institute of City University of Hong Kong, Shenzhen 518057, P. R. China.

3. Medicine center for PanorOmic Sciences, University of Hong Kong, Pokfulam, Hong Kong SAR, P. R. China.

4 Department of Thermal and Fluid Engineering, University of Twente, Enschede, 7500AE, The Netherlands.

**Materials**

**Characterization**

**Supplementary Figures**

Figure S1. (A, B) XPS of Cu₂O nanozymes.

Figure. S2. XRD of the nanozymes (Cu₂O) - initiated and crosslinked hydrogel (NICH).

Figure S3. Characterization of the mimic enzyme properties of Cu_2_O nanozymes and Cu_2_O nanozymes encapsulated in hydrogels.

Figure S4. Rheological properties of NICH.

Figure S5. Bactericidal effects of hydrophilic and hydrophobic modifications of ZnO nanowires.

Figure S6. SEM images of ZnO nanowires for (A) *E. coli* and (B) *S. aureus* lysis.

Figure S7. Characterization of the nanozymes (Cu₂O) - initiated and crosslinked hydrogel (NICH).

Figure S8. Polydispersity Index (PDI) of Cu_2_O nanoparticles and hydrogel-encapsulated nanoparticles.

Figure S9. Optical photos of detection platforms prepared by hydrogel precursor systems with different contents (25-40 μL).

Figure S10. Characterization of the NICHN 25. (A) SEM photos of NICHN 25. (B) Elemental analysis of NICHN 25

Figure S11. Fluorescent images of (A) NICH, (B) NICHN 25, (C) NICHN 30, (D) NICHN 35, and (E) NICHN 40 for treating *E. coli*, respectively

Figure S12. Fluorescent images of (A) NICH, (B) NICHN 25, (C) NICHN 30, (D) NICHN 35, and (E) NICHN 40 for treating *S. aureus*, respectively.

Figure S13. (A) Inductively Coupled Plasma Mass Spectrometry Testing for Zn²⁺ from ZnO nanowires, Cu²⁺/Cu⁺ released from NICH, and Zn²⁺, Cu²⁺/Cu⁺ released from NICHN. (B) (C) Bacterial Culture: the effects of ions released from ZnO nanowires, NICH, and NICHN on bacterial inactivation.

Figure S14. The influence of other factors on the colorimetric reaction.

Figure S15. The rheological curve of hydrogels treated with sodium chloride

Figure S16. (A) Interface illustration of the bacterial detection application on a mobile app, presenting the results for 2.8 × 10⁶ CFU of *E. coli*. (B) Reproducibility validation: tests were conducted using the same NICHN with 2.8 × 10⁶ CFU of *E. coli*. After washing and reloading *E. coli*, colorimetric assays were repeated five times.

Figure S17. Morphology and bactericidal effect of the fabricated silicon nanowires.

Figure S18. FTIR of silicon nanowires.

Figure S19. Microdroplets and their wetting and dewetting processes on silicon nanowires.

Figure S20. The bactericidal effect after modification of silicon nanowires.

Figure S21. (A) SEM image of silicon nanowires, (B) SEM image of silicon nanowires coated with NICH.

**Table S1** Comparison of different methods for the detection of bacteria.

**Materials**

Unless stated otherwise, all reagents and solvents were used as received without further purification. Zinc acetate dihydrate (Zn(CH_3_COO)_2_·2H_2_O), triethylamine ((C_2_H_5_)_3_N), isopropyl alcohol (C_3_H_8_O), sodium hydroxide (NaOH), zinc nitrate hexahydrate (Zn(NO_3_)_2_·6H_2_O), ammonium solution (NH_3_·H_2_O), hydrogen peroxide solution (H_2_O_2_) and hexamethylenetetramine (C_6_H_12_N_4_) were purchased from Sigma-Aldrich. Acrylamide (AAM), N, N′-methylene-bis-acrylamide (MBAA), and chitosan (CS) were purchased from J&K Scientific Ltd. Ascorbic acid, 50% glutaraldehyde, methanal, glutathione (GSH), and 3,3’,5,5’-tetramethylbenzidin (TMB) and CuCl_2_·2H_2_O were received from Aladdin. SYTO 9 and prodium iodide (PI) were purchased from Thermo Fisher Scientific. *E. coli*, *S. aureus* and *P. aeruginosa* were bought from ATCC (USA).

**Characterization**

SEM images and EDS mapping data were obtained by a FEI Quanta 450 FEG equipped with Oxford EDS accessories. For the observation of hydrogel structures, the samples were first freeze-dried by lyophilizer (Labconco FreeZone 71020). The size and zeta potential of CS and Cu_2_O/CS nanocomposites were measured by a Malvern Zetasizer Nano-ZS ZEN3600 using ethanol as the dispersion medium. FT-IR was measured by a PerkinElmer Spectrum 100. PXRD was recorded on a Bruker D2 Phaser diffractometer. Optical images were taken by a Nikon D5500 camera. The shear viscosity measurements were performed on a rotational rheometer (Malvern Kinexus Lab+). Tensile tests were conducted on an AMETEK TCM 100 with a 50 N force sensor. UV-vis spectroscopy was performed to obtain the optical absorption spectra and band gaps

on an Agilent Cary 4000 spectrophotometer with internal diffuse reflectance accessories.

**Supplementary Figures**


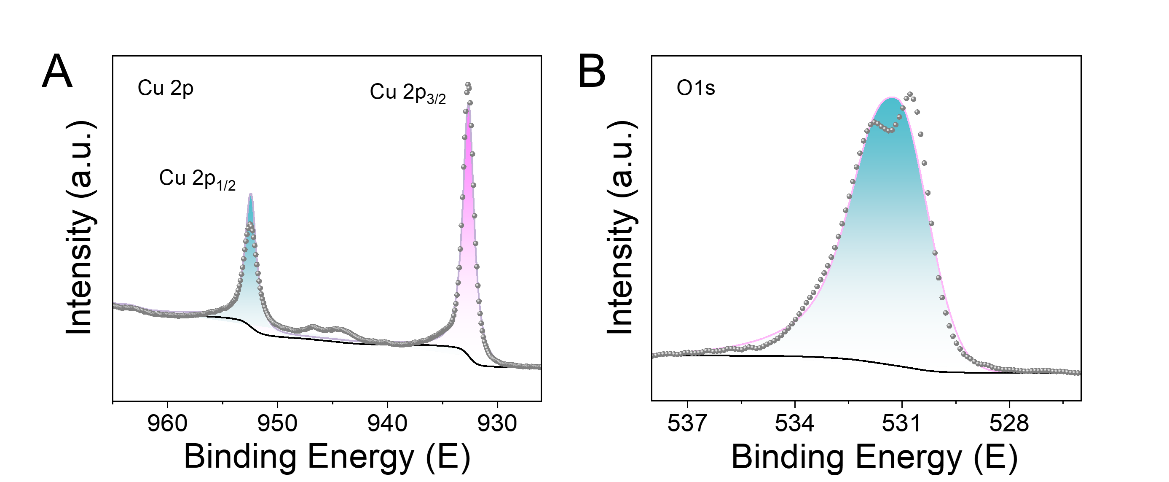


Figure S1. XPS of Cu₂O nanozymes.


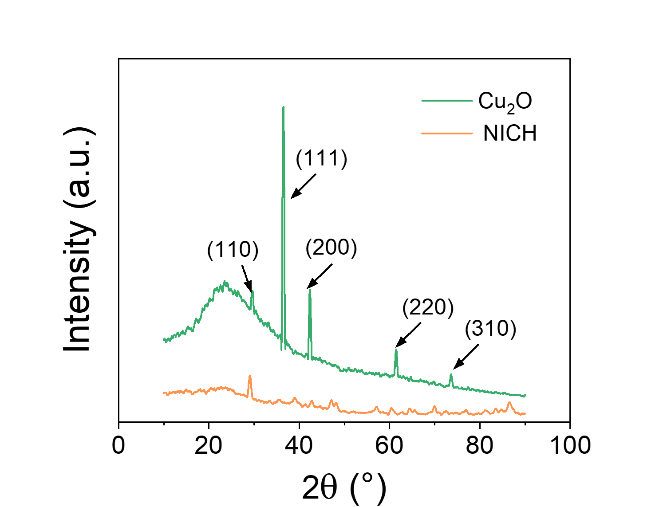


Figure S2. XRD of the nanozymes (Cu₂O) - initiated and crosslinked hydrogel (NICH).


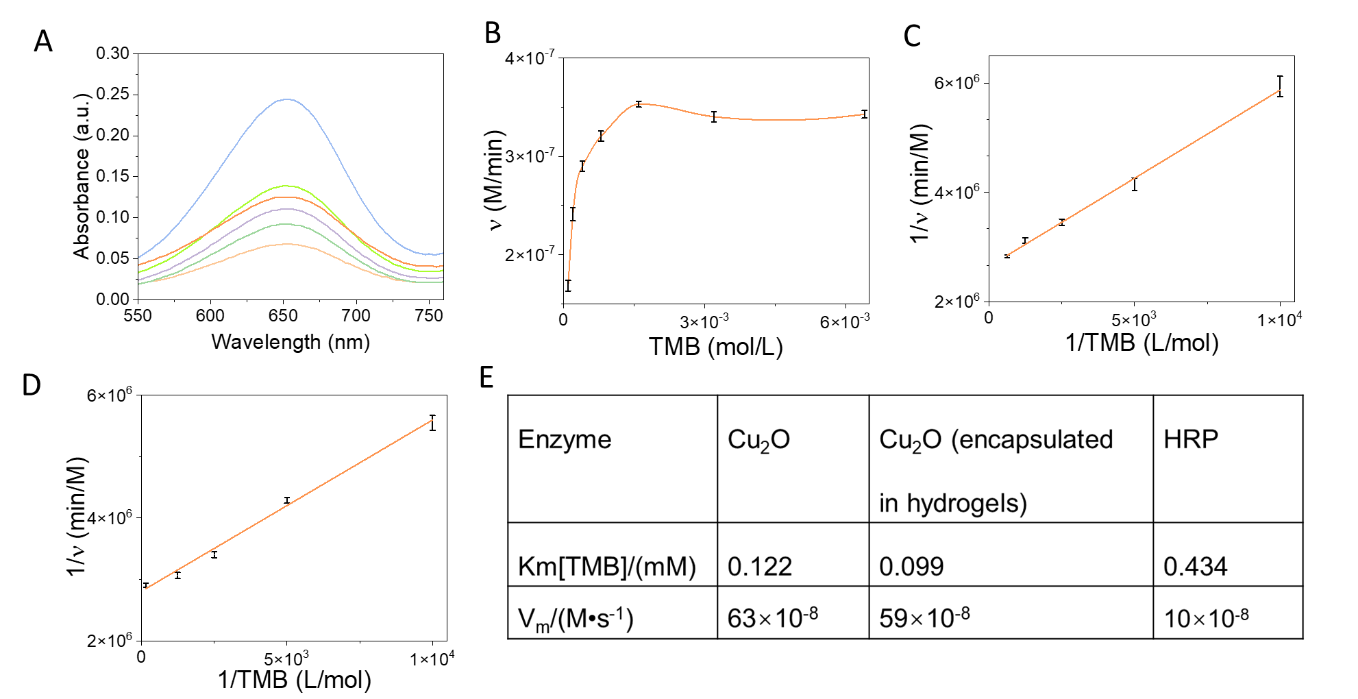


Figure S3. Characterization of the mimic enzyme properties of Cu_2_O nanozymes and Cu_2_O nanozymes encapsulated in hydrogels. (A) UV-vis absorption curves for oxidation of TMB (TMB ox) at different concentrations of TMB. (B) Enzyme kinetic curves of Cu_2_O nanozyme for TMB. (C) Double reciprocal plots of activity of Cu_2_O nanozymes. (D) Double reciprocal plots of activity of Cu_2_O nanozymes encapsulated in hydrogels. (E) Comparison of K_m_ and V_m_ for Cu_2_O nanozymes, Cu_2_O nanozymes encapsulated in hydrogels and HRP.


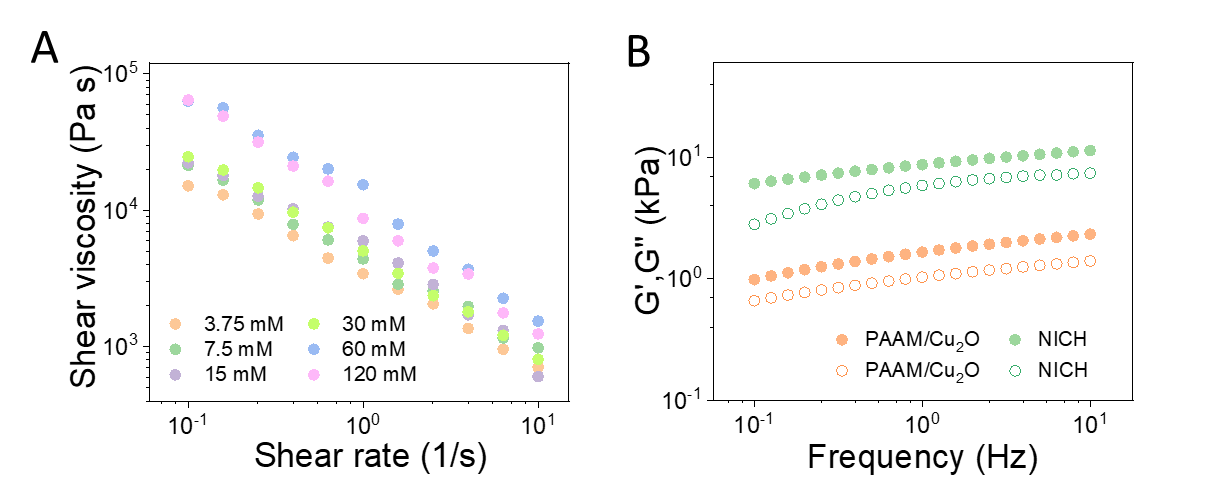


Figure S4. Rheological properties of NICH. (A) Shear viscosity of NICH formed by different content of CS. (B) Rheological properties of NICH and PAAM/Cu_2_O hydrogels.


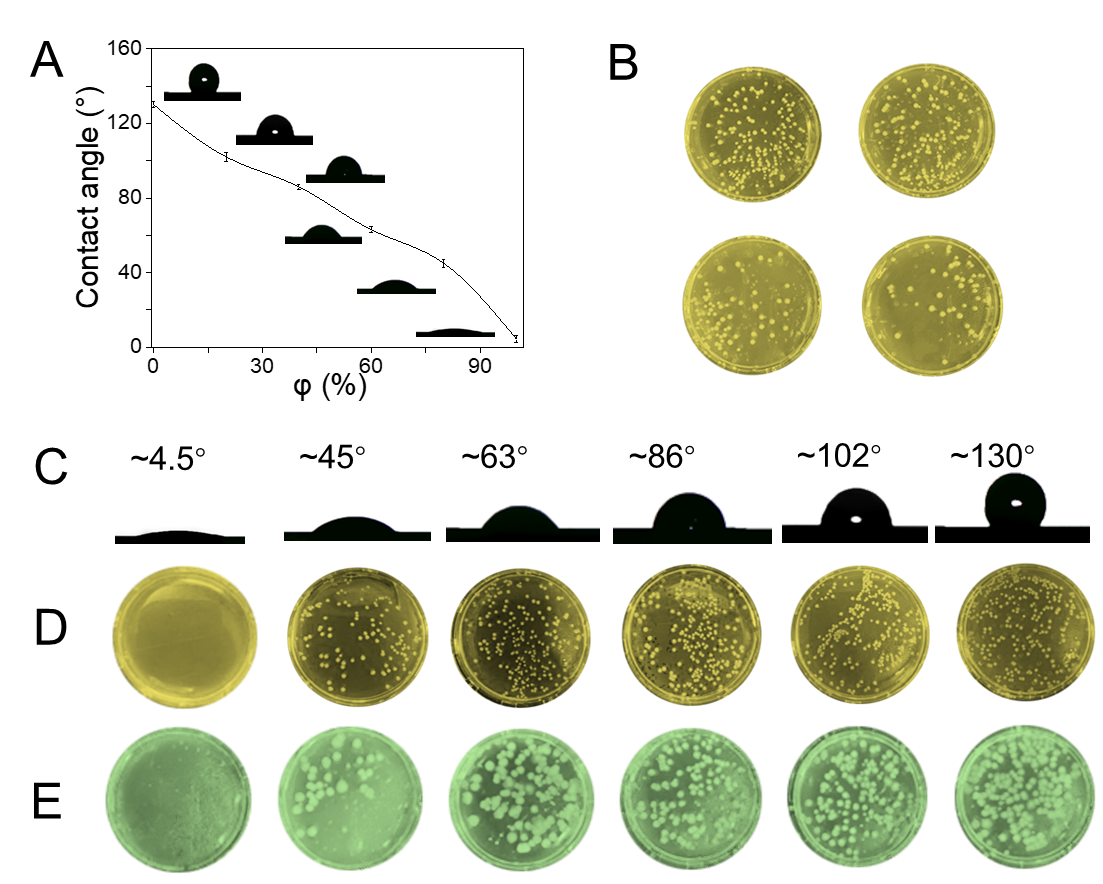


Figure S5. Bactericidal effects of hydrophilic and hydrophobic modifications of ZnO nanowires. (A) The wettability of ZnO nanowires was chemically modified, and the contact angle (θ) ranged from 4.5° to 130°. (B) Photograph of plate count agar (diameter, 60 mm) used to determine the total number of viable *S. aureus*. (C) The wettability of ZnO nanowires was chemically modified, and the contact angle (θ) ranged from 4.5° to 130°. (D) The survival rates of *E. coli* and *P. aeruginosa* (E) carried by droplets were measured using a plate counting experiment.


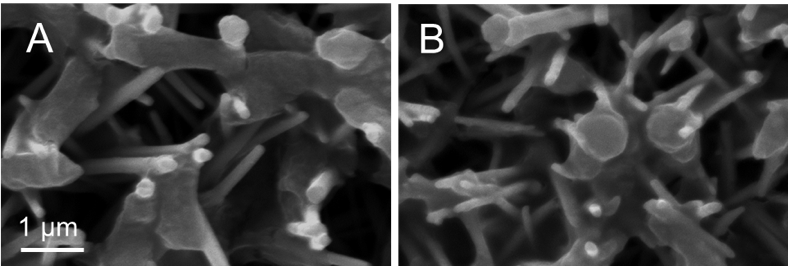


Figure S6. SEM images of ZnO nanowires for (A) *E. coli* and (B) *S. aureus* lysis.


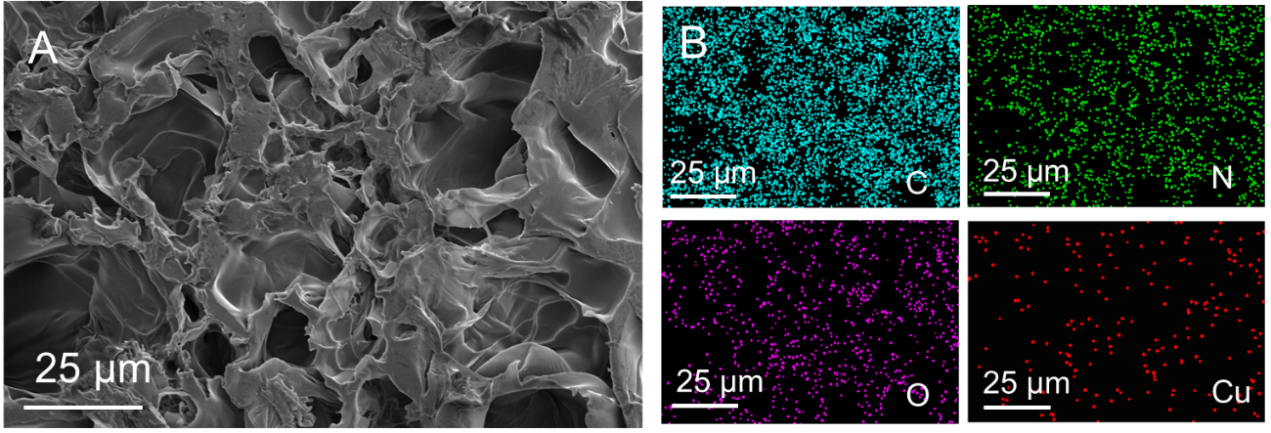


Figure S7. Characterization of the nanozymes (Cu₂O) - initiated and crosslinked hydrogel (NICH). (A) SEM photos of NICH. (B) Elemental analysis diagram of NICH.

**
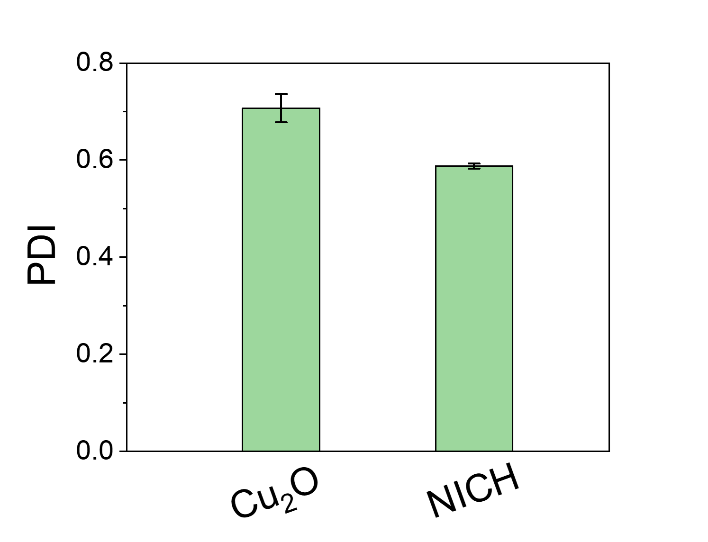
**

Figure S8. Polydispersity Index (PDI) of Cu_2_O nanoparticles and hydrogel-encapsulated nanoparticles.


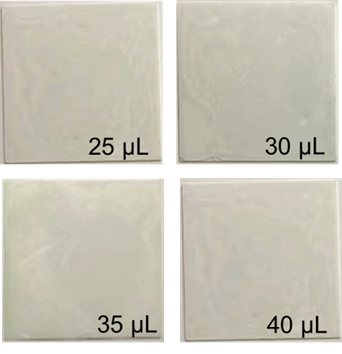


Figure S9. Optical photos of detection platforms prepared by hydrogel precursor systems with different contents (25-40 μL).


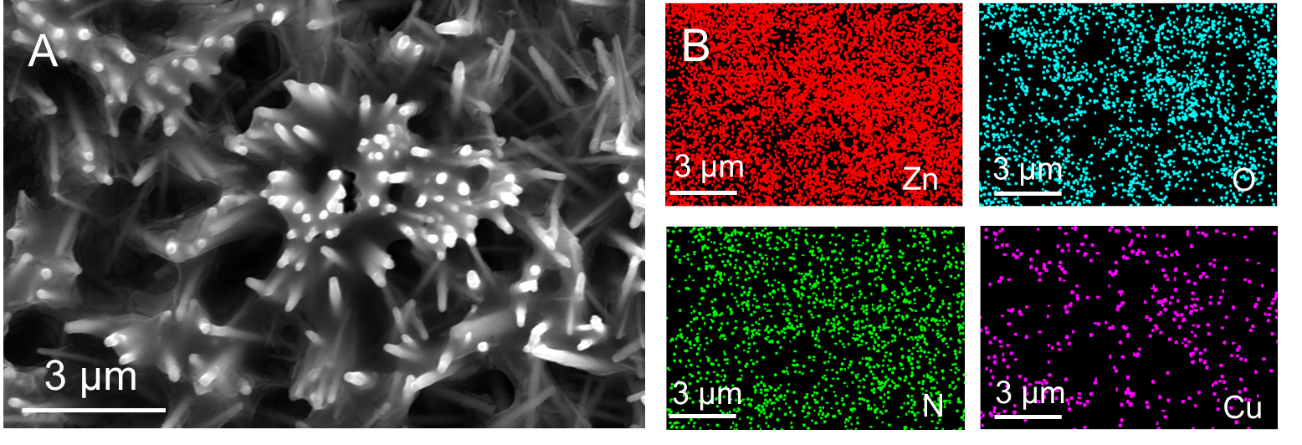
Figure S10. Characterization of the NICHN 25. (A) SEM photos of NICHN 25. (B) Elemental analysis of NICHN 25.


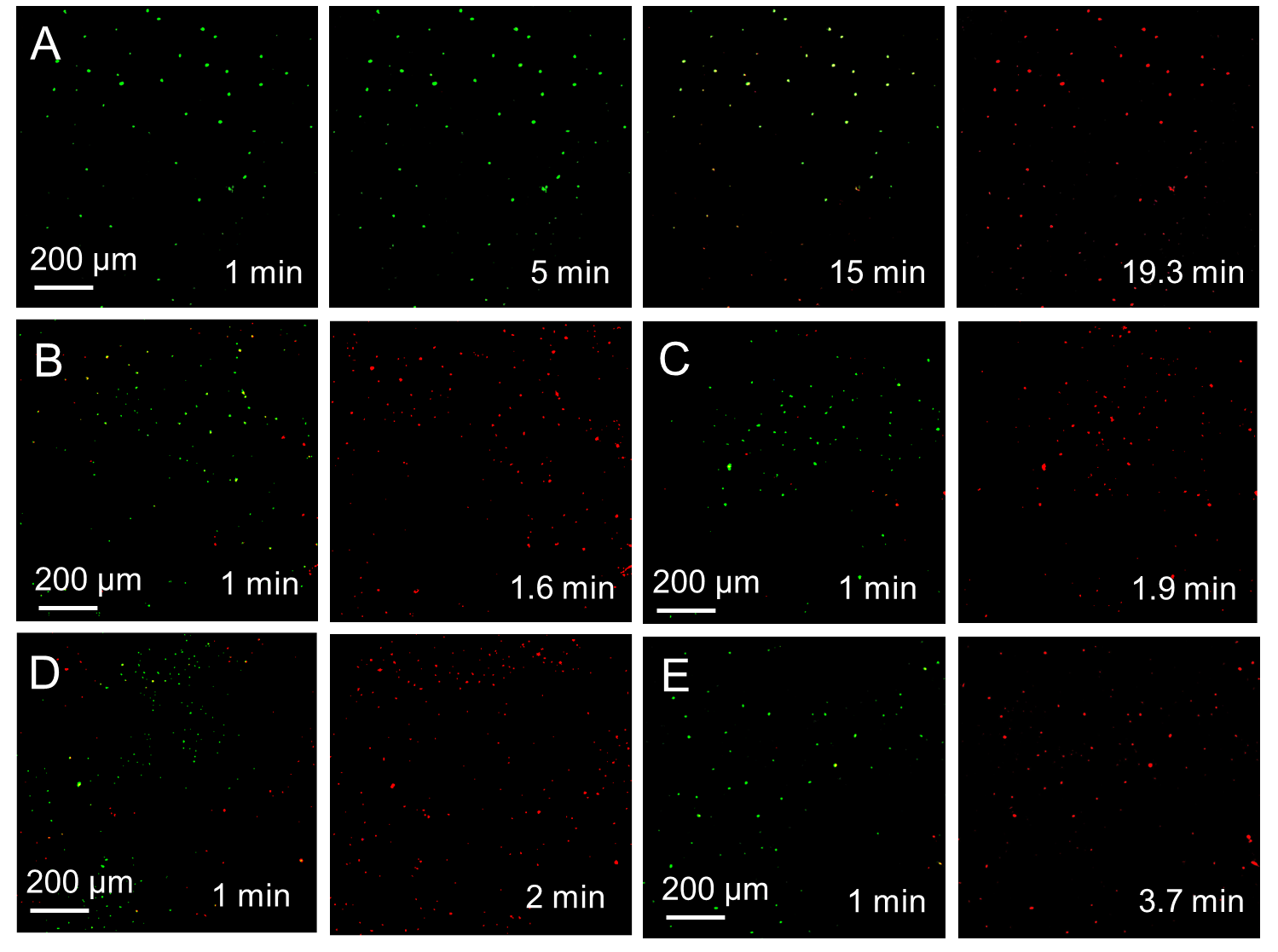


Figure S11. Fluorescent images of (A) NICH, (B) NICHN 25, (C) NICHN 30, (D) NICHN 35, and (E) NICHN 40 for treating *E. coli*, respectively.


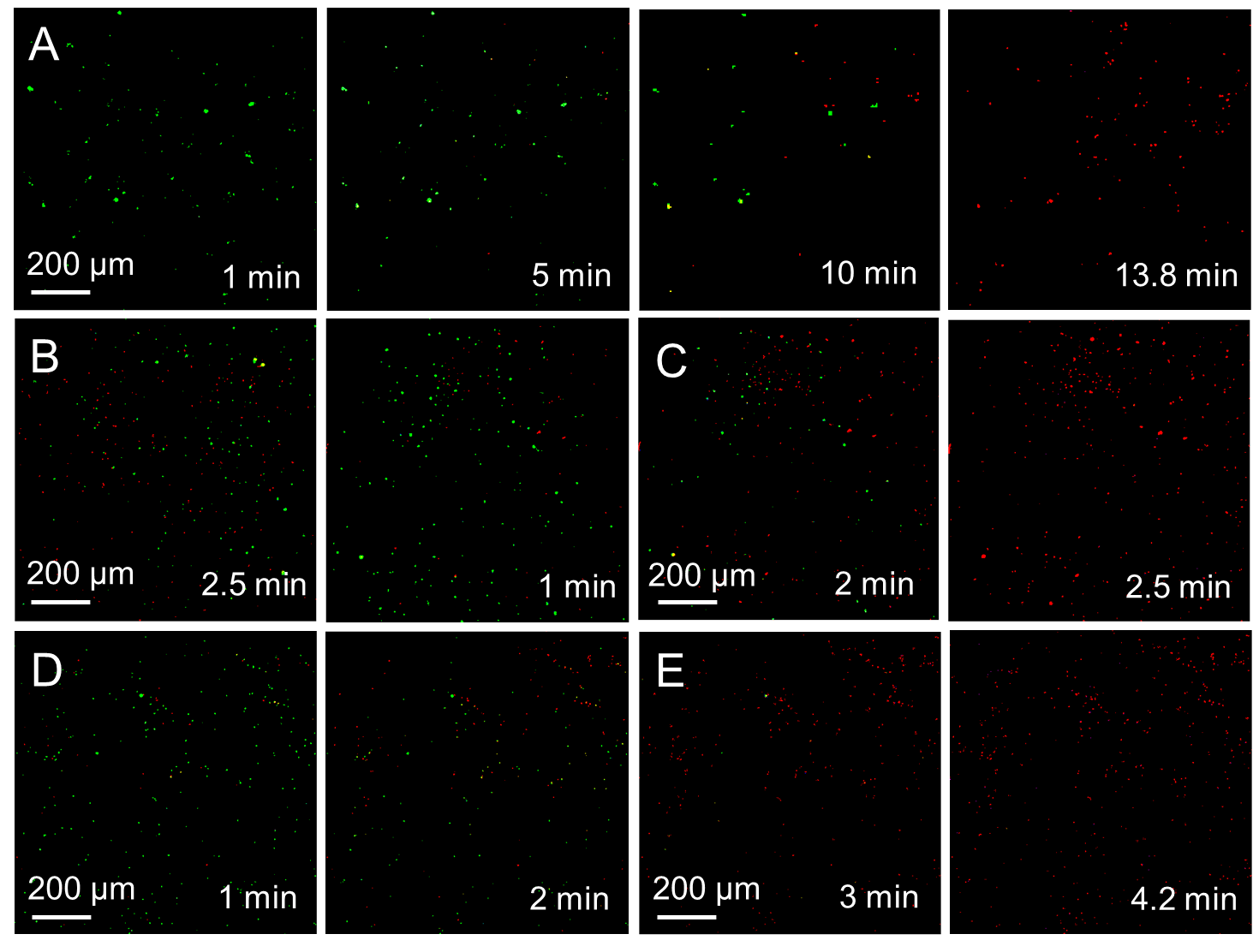


Figure S12. Fluorescent images of (A) NICH, (B) NICHN 25, (C) NICHN 30, (D) NICHN 35, and (E) NICHN 40 for treating *S. aureus*, respectively.


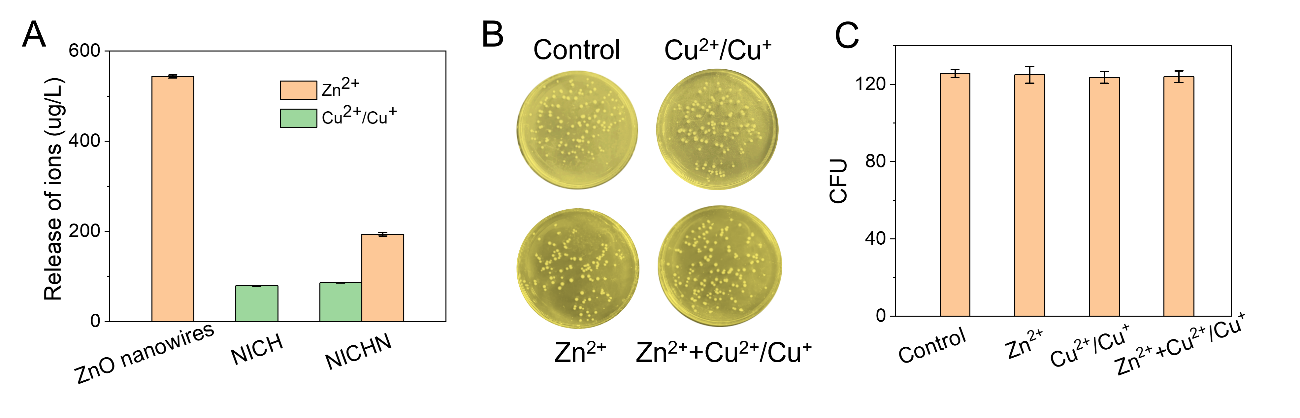


Figure S13. (A) Inductively Coupled Plasma Mass Spectrometry Testing for Zn²⁺ from ZnO nanowires, Cu²⁺/Cu⁺ released from NICH, and Zn²⁺, Cu²⁺/Cu⁺ released from NICHN. (B) (C) Bacterial Culture: the effects of ions released from ZnO nanowires, NICH, and NICHN on bacterial inactivation.


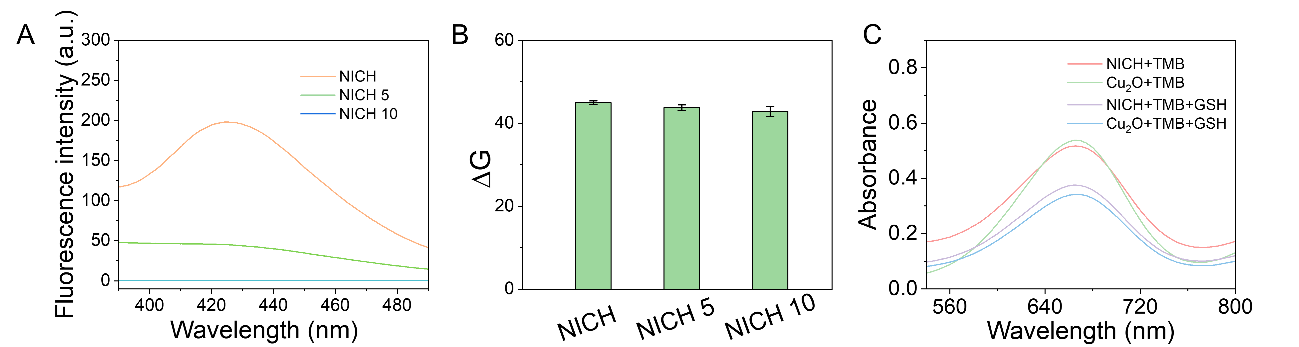


Figure S14. The influence of other factors on the colorimetric reaction. (A) Ethanol washing of the hydrogel to remove residual free radicals. NICH represents the untreated hydrogel, NICH 5 represents the hydrogel treated with ethanol for 5 minutes, and NICH 10 represents the hydrogel treated with ethanol for 10 minutes. (B) Verification of the influence of residual free radicals in the three types of hydrogels on the colorimetric assay, with corresponding changes in grayscale values. (C) The effect of hydrogel composition on the colorimetric reaction. Cu_2_O nanoparticles and NICH (encapsulating Cu_2_O nanoparticles) were added to GSH to find the influence of the blank hydrogel on the colorimetric reaction.

Figure S15. The rheological curve of hydrogels treated with sodium chloride.

**
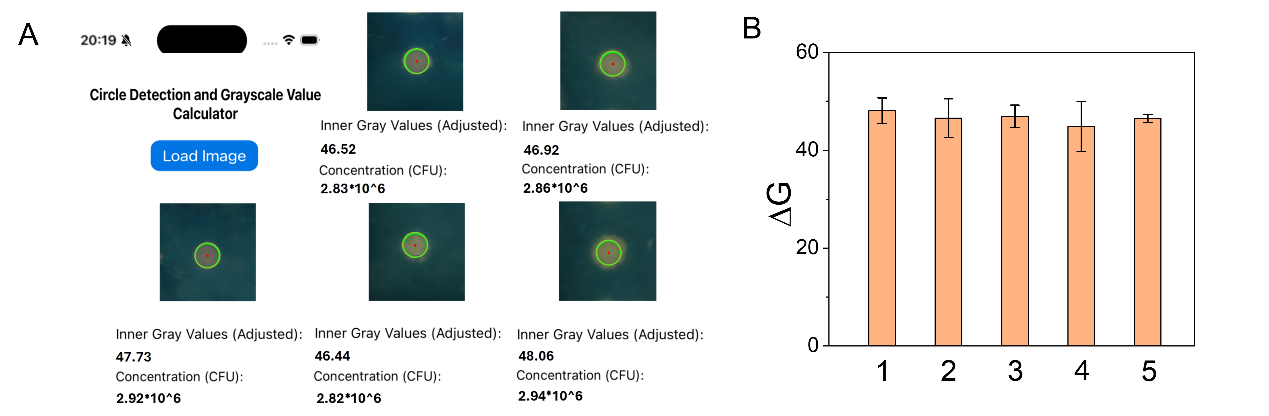
**

Figure S16. (A) Interface illustration of the bacterial detection application on a mobile app, presenting the results fo**r** 2.8 × 10⁶ CFU of *E. coli*. (B) Reproducibility validation: tests were conducted using the same NICHN with 2.8 × 10⁶ CFU of *E. coli*. After washing and reloading *E. coli*, colorimetric assays were repeated five times.


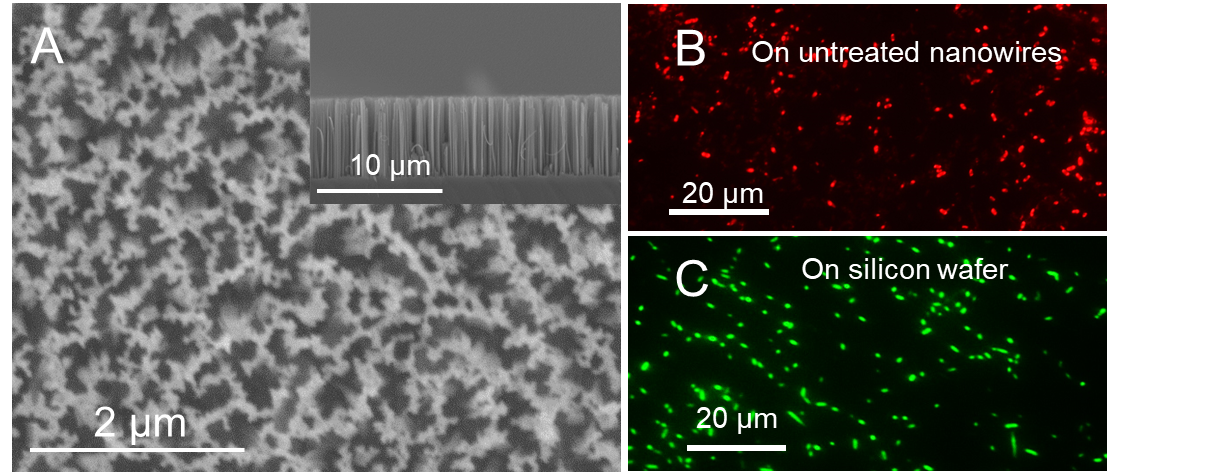


Figure S17. Morphology and bactericidal effect of the fabricated silicon nanowires. (A) Side-view and top-view SEM images of the nanowire array (*h* = 8.7±0.6 μm). (B) Fluorescent microscope imaging of dead (red) and live (green) *E. coli* after deposition from 1 μL droplet showing the bacteria killing of nanowire array in strong contrast to the control wafer (C).


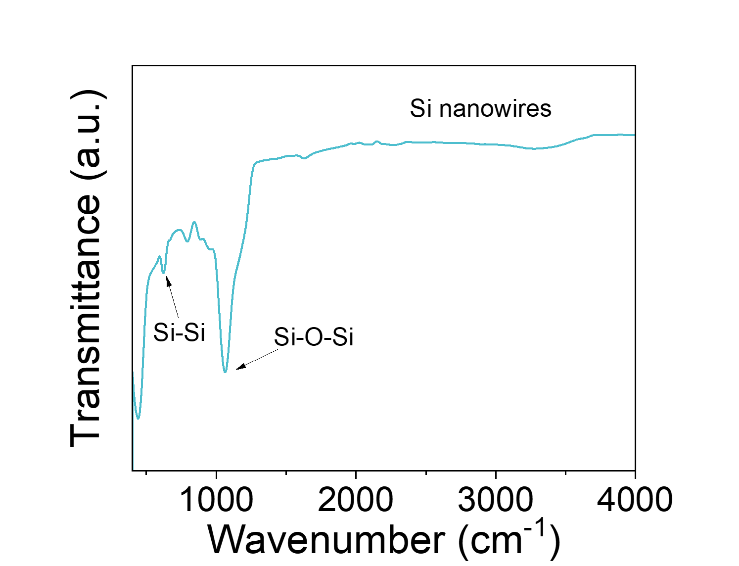


Figure S18. FTIR of silicon nanowires.


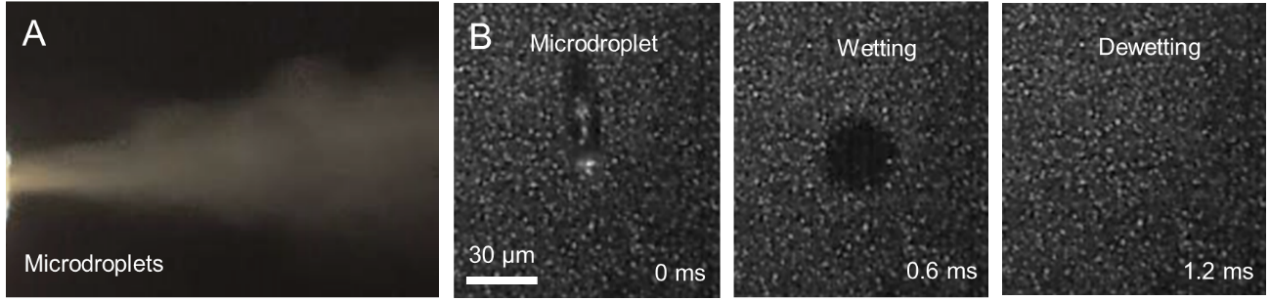


Figure S19. Microdroplets and their wetting and dewetting processes on silicon nanowires. (A) Photograph of ultrasonic atomization to generate bacteria-loaded microdroplets (diameter, 3.3 to 54.7 μm). (B) High-speed camera monitoring the microdroplet wetting and dewetting to indicate ultra-fast bacteria killing within 1.2 ms of silicon nanowires.


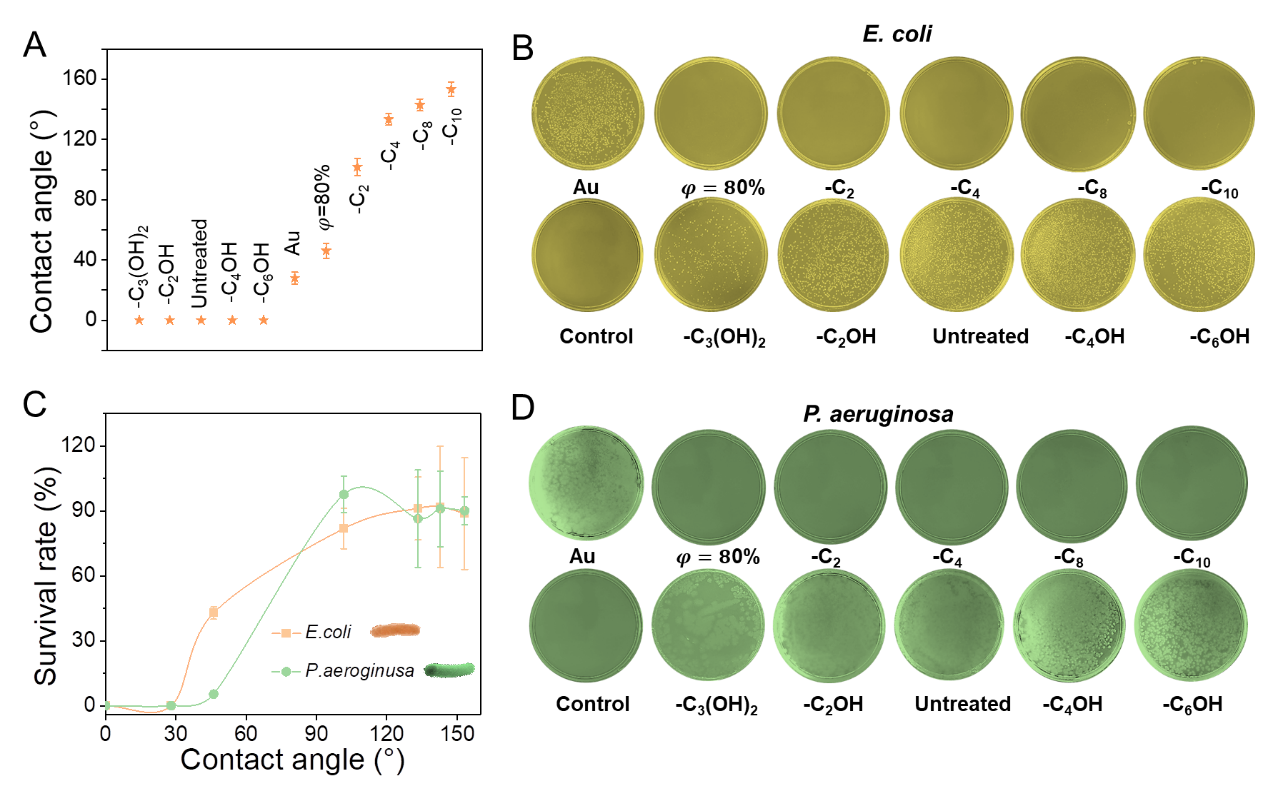


Figure S20. The bactericidal effect after modification of silicon nanowires. (A) Contact angle (*θ*) ranging from 0° to 153.3° by decorating with surface functional groups, including -C_3_(OH)_2_, -C_2_OH, -C_4_OH, -C_6_OH, Au, -C_6_OH/-C_2_ (φ = 80%), -C_2_, -C_4_, -C_8_, -C_10_ respectively, with silicon wafer as control groups. (C) Survival rates of droplet-carried *E. coli* and *P. aeruginosa* (1 μL; 10^7^ mL^-1^ CFU), respectively. Optical images of plate count agar to determine survival rate of *E. coli* (B), *P. aeruginosa* (D).


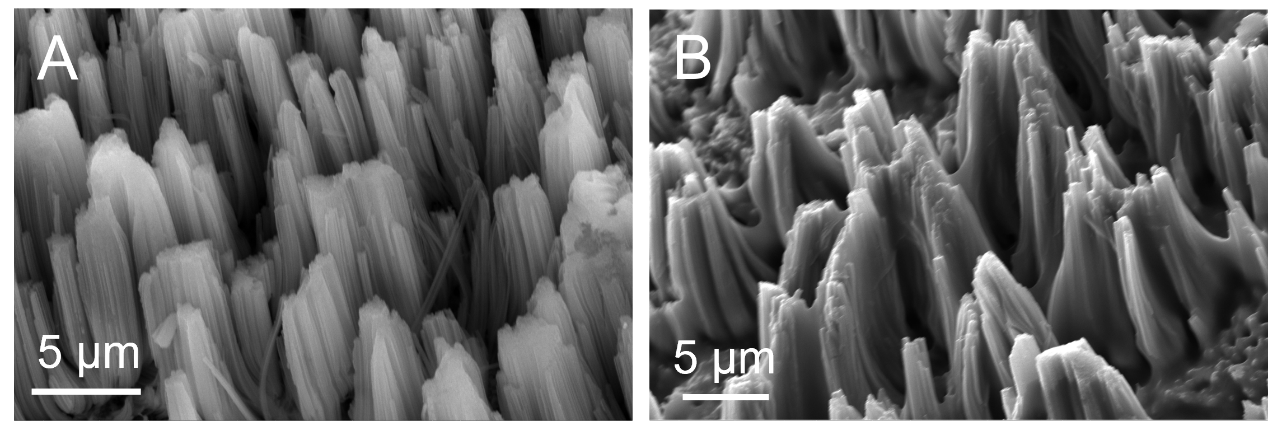


Figure S21. (A) SEM image of silicon nanowires, (B) SEM image of silicon nanowires coated with NICH.

**Table S1** Comparison of different methods for the detection of bacteria

| Bacterial inactivation | Time of whole process | Need for antibodies or aptamers | Need for complex equipment | Reference |
| --- | --- | --- | --- | --- |
| Yes | 38 min | No | Automatic microplate reader | ^[^[^1^](#_ENREF_1)^]^ |
| Yes | 15 min | Yes | The 808 nm laser | ^[^[^2^](#_ENREF_2)^]^ |
| Yes | 30 min | Yes | Fluorescence Spectrometer | ^[^[^3^](#_ENREF_3)^]^ |
| No | 18 min | Yes | Surface-Enhanced Raman Spectroscopy Spectrometer | ^[^[^4^](#_ENREF_4)^]^ |
| Yes | 21 min | No | No | This work |

REFERENCES

[1] Y. Chen, M. Zhang, D. Lu, J. Yin, W. Gao, X. Shi, Capture, Sterilization and Determination Platform of Foodborne Pathogen Based on Halbach Ring-Mediated Magnetic Filter, *Sens. Actuators, B* **2023**, 388, 133844.

[2] S. Liu, R. Shu, H. Jia, K. Wang, B. Wang, J. Zhang, J. Sun, N. Sattorov, K. B. Makhmudov, M. Jin, Antibody-Level Bacteria Grabbing by “Mechanic Invasion” of Bioinspired Hedgehog Artificial Mesoporous Nanostructure for Hierarchical Dynamic Identification and Light-Response Sterilization, *Adv. Mater.* **2025**, 2416906.

[3] J. Feng, J. Li, X. Lin, L. Kang, N. Duan, Z. Wang, S. Wu, A Polydopamine Coated Magnetic Spiral Switchable Composite Material For Photothermal Sterilization and Dual-color Fluorescence Detection of Food-Borne Pathogens, *Sens. Actuators, B* **2025**, 425, 136935.

[4] J. Li, W. Shen, X. Liang, S. Zheng, Q. Yu, C. Wang, C. Wang, B. Gu, 2D Film-Like Magnetic SERS Tag with Enhanced Capture and Detection Abilities for Immunochromatographic Diagnosis of Multiple Bacteria, *Small* **2024**, 20, 2310014.
